# Multifunctionality of prostatic acid phosphatase in prostate cancer pathogenesis

**DOI:** 10.1042/BSR20211646

**Published:** 2021-10-22

**Authors:** Evgenia Alpert, Armin Akhavan, Arie Gruzman, William J. Hansen, Joshua Lehrer-Graiwer, Steven C. Hall, Eric Johansen, Sean McAllister, Mittul Gulati, Ming-Fong Lin, Vishwanath R. Lingappa

**Affiliations:** 1 Bioconformatics Laboratory of the California Pacific Medical Center (CPMC) Research Institute, 475 Brannan St, SF, CA 94107, U.S.A.; 2 Prosetta Corporation, 670 5th St, SF, CA 94107, U.S.A.; 3 Cell and Tissue Biology, UCSF, 513 Parnassus Ave, SF, CA 94143, U.S.A.; 4 Biomolecular Resource Center Mass Spectrometry Facility, UCSF, 513 Parnassus Ave, SF, CA 94143, U.S.A.; 5 Cancer Center, UCSF, 513 Parnassus Ave, SF, CA 94143, U.S.A.; 6 Cancer Laboratory of the California Pacific Medical Center (CPMC) Research Institute, 475 Brannan St, SF, CA 94107, U.S.A.; 7 Department of Physiology, UCSF, 513 Parnassus Ave, SF, CA 94143, U.S.A.; 8 Department of Biochemistry and Molecular Biology, University of Nebraska Medical Center, Omaha, NE 68198, U.S.A.

**Keywords:** androgen sensitivity, human prostatic acid phosphatase, prostate cancer, signal sequence

## Abstract

The role of human prostatic acid phosphatase (PAcP, P15309|PPAP_HUMAN) in prostate cancer was investigated using a new proteomics tool termed signal sequence swapping (replacement of domains from the native cleaved amino terminal signal sequence of secretory/membrane proteins with corresponding regions of functionally distinct signal sequence subtypes). This manipulation preferentially redirects proteins to different pathways of biogenesis at the endoplasmic reticulum (ER), magnifying normally difficult to detect subsets of the protein of interest. For PAcP, this technique reveals three forms identical in amino acid sequence but profoundly different in physiological functions, subcellular location, and biochemical properties. These three forms of PAcP can also occur with the wildtype PAcP signal sequence. Clinical specimens from patients with prostate cancer demonstrate that one form, termed ^PL^PAcP, correlates with early prostate cancer. These findings confirm the analytical power of this method, implicate ^PL^PAcP in prostate cancer pathogenesis, and suggest novel anticancer therapeutic strategies.

## Introduction

Many aspects of prostate cancer pathogenesis remain a mystery, including the early triggers for malignant transformation and the basis for progression to androgen independence, two critical milestones in the natural history of prostate cancer [1]. Our studies suggest a remarkable and unexpected relationship between these milestones in prostate cancer and different folded forms of prostatic acid phosphatase (PAcP).

PAcP has long been recognized to be important in prostate cancer, with studies demonstrating it to be a critical, androgen-dependent regulator of prostatic epithelial cell growth [2–6]. However, heterogeneity of subcellular location, and confounding functional associations, have made a full understanding of its role in physiology and disease challenging. The growth regulatory form of PAcP appears to be intracellular [7–9]. Yet, a major fraction of PAcP is found secreted into seminal fluid, where it likely has other functions [10]. In early prostate cancer, PAcP is secreted into the bloodstream [11]. Later, with prostate cancer progression, PAcP expression by the cancer is typically lost, limiting its value as a clinical epidemiological biomarker, a use that has been supplanted by prostate-specific antigen (PSA) [12].

Previous studies have determined that diverse pathways of protein biogenesis at the endoplasmic reticulum (ER) allow some proteins to achieve substantial heterogeneity [13–16], in some cases, with distinctive functions [17]. By analogy, we hypothesized that intracellular, bloodstream, and seminal fluid forms of PAcP represented the outcomes of different pathways of biogenesis, resulting in forms that may be responsible for different functions, some of which could contribute to prostate cancer. A challenge to testing this hypothesis is that functionally distinct subsets of proteins, including PAcP, may be extremely difficult to detect and distinguish among the plethora of post-translational modifications (PTMs) occurring *in vivo.* Moreover, a host of confounders can mask or render ambiguous otherwise clear cut distinctions between protein subsets. For example, one form of a protein may be more flexible, exposing a buried epitope transiently, thereby mimicking another very differently folded form in which that epitope is always on the surface. As a result, even an epitope-specific antibody would fail to discriminate clearly between them.

A recent discovery suggested a novel way to deal with such difficulties. It was found that, in addition to targeting nascent secretory and membrane proteins to the membrane of the ER [18], amino terminal cleaved signal sequences can also determine the pathway(s) of biogenesis of secretory and membrane protein [14,15]. Thus the distribution between functionally distinct forms of prion protein (PrP) achieved at the ER could be altered, either biologically (by changes in the *trans*-acting proteins with which the nascent chain interacts) [13,19] or artificially (by swapping the native signal sequence of PrP for signal sequences of other subtypes [14,18,20]. If the N-terminal signal sequence is correctly cleaved, the family of mature proteins generated from the identical nascent chain behind different signal sequences will be identical in primary sequence. However, subsets of those chains may differ in either folding and/or PTMs, particularly if they have been made via different pathways at the ER. Depending on the signal sequence chosen, a minor population of folded or modified chains can have its expression magnified, and therefore can be more readily characterized, particularly because regulatory mechanisms that might mask a given form (e.g. by feedback down-regulation) will have been bypassed (Supplementary Figure S1).

## Results

To test our hypothesis above, we wished to determine first, whether mature PAcP expressed behind domains of different subtypes of signal sequences, resulted in differences in functional phenotype, and if so, to then correlate those to differences in subcellular location and biochemical characteristics.

The signal sequences chosen were those of immunoglobulin G heavy chain (IgG) and prolactin (Prl), because they have been previously shown to favor very different pathways of biogenesis by a clearly demonstrable mechanism [14,15]. The IgG signal sequence directs formation of an ‘open’ ribosome–membrane junction (RMJ) that allows the nascent chain to transiently interact directly with the cytosol, despite ultimately residing in the ER lumen upon chain completion [10,21]. In contrast, the Prl signal sequence directs formation of a ‘closed’ RMJ whereby the nascent chain is shielded from the reducing environment of the cytosol and exposed to the oxidizing environment of the ER lumen [14,15,22]. Differences in character of the RMJ have been defined by accessibility of the nascent chain to exogenously added probes such as proteases and antibodies [21,22]. These differences provide plausible explanations for how a change in signal sequence could result in a change in the nascent chain’s pathway of biogenesis, and therefore, a change in the mixture of functional forms synthesized, despite primary amino acid sequence identity of the mature protein [13–15].

### Use of chimeric signal sequences avoids the risk of miscleavage

**Figure 1:**
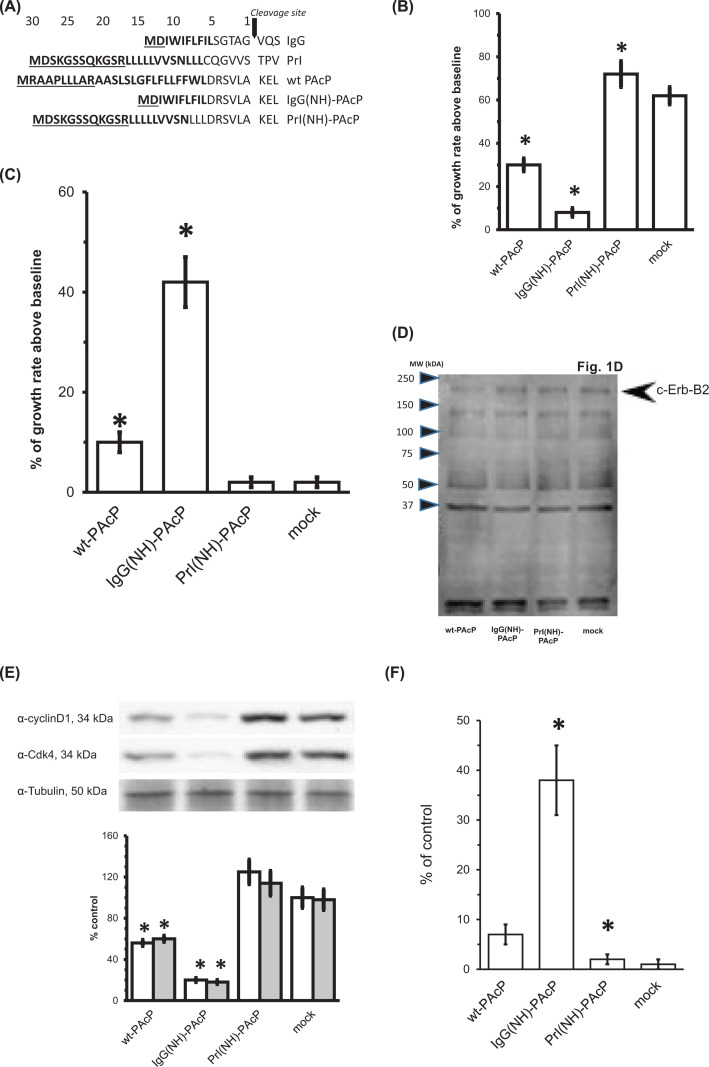
Cellular effects of the PAcP and its chimeric signal sequence constructs in LNCaP cells (**A**) **Line diagram of wtPAcP, IgG and Prl signal sequences.** N (bold and underlined) and H (bold without underline) domains have been engineered in front of the C domain of the wt PAcP signal sequence. Numbers above are amino acids of the signal sequence preceding the cleavage site. Cleavage site is indicated with downward pointing arrow, followed by three residues of the mature protein sequence. (**B**) **Growth rate of LNCaP clone C81 cells transfected with PAcP and chimeric signal sequence constructs.** Two sets of cells were transfected with pcDNA3.1zeo+ vector harboring full-length human PAcP and chimeric signal sequence constructs, as described in ‘Methods’ section. Mock represents cells transfected with empty pcDNA3.1zeo+ vector. The first set of cells was harvested and then counted immediately after transfection (5 h) and this number was used as the baseline for growth rate determination. The second set was harvested and counted 72 h after transfection. Data shown are % of growth rate above baseline. Mean ± S.D. (*n*=7); *P<0.03, significantly different from control (C81). (**C**) **Growth rate of LNCaP clone C81 cDNA transfected cells in present of androgen.** Two sets of LNCaP C-81 cells were transfected as for (B). The first set was harvested and counted 72 h after transfection and cell number used as a baseline for growth rate. A total of 10 nM of DHT was added to the other set for the last 48 h of transfection and then cells harvested and counted. Data shown are % of growth rate above baseline at 72 h. Mean ± S.D. (*n*=5); *P<0.04, significantly different from control (C81). (**D**) **Effect of the addition of androgen on the level of c-Erb-B2 (pp185) in different types of transfected cells.** Cells were transfected and androgen was added to the medium as described in (C). Seventy-two hours after transfection cells were harvested, lysed, and equal amounts of total protein (50 µg) were electrophoresed on SDS/PAGE for WB analysis with the anti-pY clone 4G10. Leftward pointing arrowhead indicates position of c-Erb-B2 (pp185). (**E**) **Cell growth arrest at G_0_/G_1_ phase when transfected with IgG(NH)PAcP.** Cells transfected as described were harvested, lysed, and equal amount of total proteins analyzed by SDS/PAGE and WB with polyclonal antibody reactive to cyclin D (open bars) or Cdk4 (closed bars). Lysates were probed with anti tubulin as a loading control shown. Data are mean ± S.D. (*n*=4); *P<0.03, significantly different from control (C81). (**F**) **Addition of Prl(NH)PAcP transfected cell medium overcomes androgen dependence of IgG(NH)PAcP transfected cells.** Two sets of LNCaP clone C81 cells were transfected with Prl(NH)-PAcP or empty vector. Seventy-two hours after transfection medium from both sets was harvested and used as a replacement medium for another two sets of cells (transfected as indicated) upon completion of transfection (5 h). Seventy-two hours later, cells were harvested, counted. The baseline for calculation of percent growth was taken to be growth in the presence of C81 (mock transfection) medium. A control set of cells was transfected as indicated at the same time without replacement media. The number of cells in that control was not statistically significantly different from cells that received mock replacement medium. Data shown are % of growth rate above baseline. Mean ± S.D. (*n*=4); *P<0.01, significantly different from control (C-81). Abbreviations: DHT, 5-alpha dihydrotestosterone; SDS/PAGE, polyacrylamide gel electrophoresis in sodium dodecyl sulfate; WB, Western blotting.

One concern to us was that a clean swap of signal sequences might result in miscleavage by signal peptidase, whose substrate recognition is strongly influenced by the residues of the cleavage site [23]. If the cleavage site was to change, a new amino terminus would be generated, confounding the interpretation of any functional differences observed, because the mature chains would no longer be identical in primary sequence, and the difference might be an artifact of the experimental method (signal sequence swapping). To avoid this possibility, only the amino terminal (N) and hydrophobic (H) domains, rather than the entire signal sequence were swapped, leaving, in all cases, a common carboxy (C) terminal signal sequence domain and cleavage site, as found in wt-PAcP. Previous studies have shown that the N and H domains are sufficient to confer the swapped signal sequence’s phenotype with respect to pathway of biogenesis [14,15]. Thus, selective domain swapping, generating chimeric signal sequence coding regions in which the C terminal domain of the signal sequence is not altered, ensures conservation of the cleavage site with that of the wildtype protein, (see Figure 1A). The construct encoding wildtype PAcP with the signal sequence N and H domains exchanged for those of IgG, will henceforth be referred to as IgG(NH)-PAcP. Likewise, the construct containing the Prl signal sequence N and H domains exchanged for those of the PAcP signal sequence will be referred to as Prl(NH)-PAcP. The fidelity of signal peptidase cleavage in both cases has been confirmed by mass spectrometry as will be discussed below.

The working hypothesis we initially explored is that IgG(NH)-PAcP favors biogenesis of ^GR^PAcP, a form that renders prostatic epithelial growth androgen sensitive, while Prl(NH)-PAcP favors biogenesis of ^PL^PAcP, a form that is a growth factor. Both of these forms are hypothesized to be different from ^SF^PAcP, the form predominant in seminal fluid. Indicated constructs were subcloned into expression vectors behind the SP6 and CMV promoters for expression in cell-free translation systems and transfected mammalian cells, respectively. Expression in cell-free systems confirmed that the heterologous chimeric signal sequences functioned with respect to targeting to, and translocation across, the ER membrane, and resulted in signal sequence cleavage and N-linked glycosylation, (see Supplementary Figure S2). That the chimeric signal sequences containing IgG and Prl N and H domains formed ‘open’ and ‘closed’ RMJs, respectively, on PAcP as observed previously for other proteins [14,15], was confirmed (Supplementary Figure S3).

### Expression of IgG(NH)-PAcP magnifies growth arrest

LNCaP clone C81 is a human prostate cancer cell line that no longer expresses PAcP and is androgen-independent for growth [24,25]. These cells were transfected with constructs encoding wtPAcP, IgG(NH)-PAcP, Prl(NH)-PAcP, and empty vector (mock transfection). Figure 1B displays the quantified results of cell proliferation 72 h after transfection. As can be seen, IgG(NH)-PAcP results in a dramatic growth arrest (>80% inhibition) compared with mock transfected cells. Green fluorescent protein (GFP) transfection demonstrated that the efficiency of transfection under the conditions used, was approximately 80–95% (data not shown). Transfection of wtPAcP has a more modest effect, with approximately 50% growth inhibition. In contrast, transfection with Prl(NH)-PAcP actually *stimulated* growth to levels significantly greater than C81 cells transfected with empty pcDNA vector. Measurement of the precise magnitude of growth stimulation was confounded by the growth-inhibiting effect of cell confluence on the maximally growth stimulated Prl(NH)-PAcP transfected cells. The growth stimulation effect was addressed more precisely by other experiments to be discussed below. Growth inhibition was corroborated by experiments involving ^3^H thymidine incorporation to measure DNA synthesis, and ^3^H leucine incorporation to measure protein synthesis, both of which were correspondingly diminished for IgG(NH)-PAcP (data not shown), consistent with Figure 1B.

### Renewal of androgen sensitivity upon IgG(NH)-PAcP expression

Given the importance of hormone sensitivity and resistance in prostate cancer pathogenesis, we wished to understand whether the apparent growth-inhibitory effect of transfection with IgG(NH)-PAcP might reflect return of androgen-independent LNCaP C-81 cells to an androgen-dependent state. To evaluate this possibility, cells were provided with androgen-supplemented medium (Figure 1C). Remarkably, the growth-inhibited IgG(NH)-PAcP transfected cells displayed massive growth stimulation upon addition of androgen, approximating the growth observed for androgen-independent, mock transfected LNCaP C-81 cells. Wt-PAcP transfected cells showed a modest proliferative response, but neither mock transfected or Prl-transfected cells showed any significant change in growth response to androgen, compared with parallel transfections containing non-androgen-supplemented medium. Since addition of androgen dramatically stimulated their growth, the inhibition of growth observed for IgG(NH)PAcP-transfected LNCaP C81 cells is consistent with re-entrainment to an androgen-sensitive state. No differences in the level of androgen receptor expression were seen by Western blotting (WB) (data not shown).

To explore the mechanism of renewal of androgen sensitivity of growth, transfected cells were analyzed for the phosphorylation state of key growth-regulatory proteins in the ErbB-related signaling cascade by which classical androgen-sensitive prostatic epithelial growth is known to occur [26]. It is shown by WB with a phosphotyrosine-specific monoclonal antibody (mAb) (Figure 1D) that upon addition of androgen, phosphorylation of c-Erb-B2 p185 appears almost equal in two types of the transfected cells (IgG(NH)-PAcP and Prl(NH)-PAcP), but not in the wtPAcP (Figure 1D). We assumed that c-Erb-B2 is the band in the 185-kDa area in the gel. In addition, medium from the Prl(NH)-PAcP transfected cells activates the ErbB-related signaling pathway, but in the absence of androgen, as shown in Supplementary Figure S4A, functional consequence of c-Erb-B2 phosphorylation is downstream phosphorylation of Shc p52 [36,37], specifically on Tyr^317^, which was explored in the growth-arrested and androgen-restored IgG(NH)-PAcP transfected cells and found to corroborate the conclusions from analysis of p185 phosphorylation (Supplementary Figure S5).

The nature of the growth-inhibiting effect was further explored by use of cyclin D1/2-specific antibodies [27]. We observed that cyclins D1/2 and Cdk4 were significantly down-regulated in IgG(NH)PAcP and wt PAcP transfected C-81 cells, approximately 70 and 30%, respectively, compared with either Prl(NH)PAcP transfected and mock transfection, despite comparable expression of the marker protein tubulin (see Figure 1E). This finding is consistent with the expectation that IgG(NH)-PAcP inhibits growth via cell cycle arrest in G_0_/G_1_ phase [28,29]. Thus, multiple lines of evidence suggest that the growth-inhibiting effect of IgG(NH)-PAcP expression is due to reversal of androgen independence of the parental LNCaP C81 cell line, and reflects suppression of the known androgen-dependent ErbB growth signaling pathway in prostatic epithelial cells.

### An alternate growth-promoting function enhanced by Prl(NH)-PAcP

To better explore the suggestion of a different function (cell proliferation rather than growth arrest) upon expression of Prl(NH)PAcP (see Figure 1B), we established a more robust proliferative assay. We found that addition of medium from Prl(NH)PAcP transfected cells, to growth-arrested IgG(NH)PAcP transfected cells (whose growth was shown in Figure 1C to be exquisitely androgen dependent), resulted in growth stimulation in the *absence* of added androgen (Figure 1F). No such effect was observed from mock transfection medium (the baseline for Figure 1F), and the effects paralleled those of androgen (Figure 1C). When analyzed as previously for IgG(NH)-PAcP, it is apparent that the same ErbB pathway is engaged, but in a different (androgen independent) and opposite (constitutively phosphorylated) manner (Supplementary Figure S5). L(+)-tartrate affinity chromatography was subsequently used to purify ^PL^PAcP directly corroborate its role as a growth factor (data not shown).

**Figure 2:**
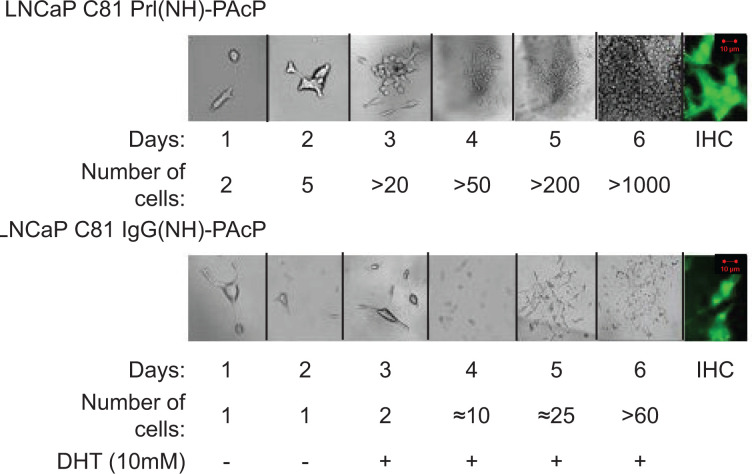
Stable cell lines confirm observations from transient transfection with IgG(NH)PAcP and Prl(NH)PAcP LNCaP clone C-81 cells were transfected as previously described in Figure 1. Stable cell lines were selected with 0.35 mM zeocin. Expression of PAcP was confirmed by WB and ICC. Cells were observed and counted daily for 6 days, with androgen added to cells at day 3. Abbreviation: ICC, immunocytochemistry.

Stable cell lines were generated to recapitulate the conclusion from transient transfection (Figure 2). Stable LNCaP C-81 derived cell lines expressing IgG(NH)-PAcP resulted in androgen-dependent growth (Figure 2, lower panel). In the absence of androgen no proliferation was observed for up to 10 days (data not shown). Upon addition of androgens, dramatic proliferation occurred (Figure 2, lower panel). In contrast, the stable cell lines expressing Prl(NH)-PAcP rapidly proliferated in the absence of added androgens (Figure 2 upper panel). Thus stable cell lines recapitulate the conclusions from transient transfection, with regard to the utility of signal sequence swapping for revealing differences in PAcP function.

**Figure 3:**
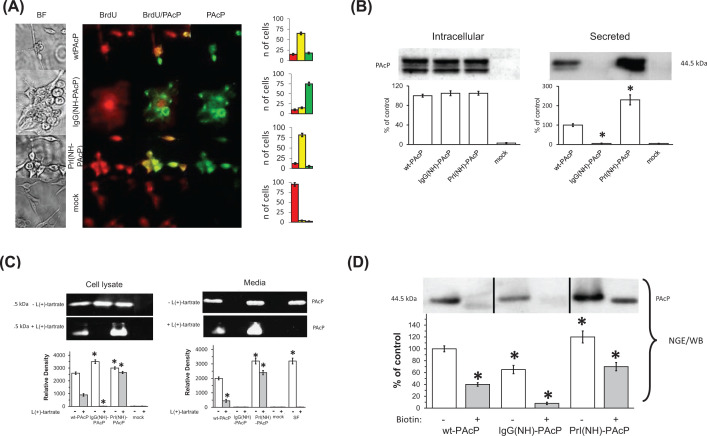
A correlation between topology and biological activity of PAcP and its chimeric signal sequence constructs (**A**) ICC staining for BrdU incorporation and PAcP expression. LNCaP clone C-81 cells were plated on a slide and transfected as indicated in Supplementary Methods. Forty-eight hours after transfection cells were incubated with 10 µM BrdU at 37°C for 1 h, washed, fixed with 95% ethanol 5% acetic acid, and blocked with 5% BSA for 1 h. Cells were subsequently incubated with anti-BrdU [2B1] mAb followed by secondary anti-mouse antibody conjugated to cyanin CY3. Then cells were washed and incubated with polyclonal anti-PAcP antibody followed by incubation with secondary anti-rabbit antibody conjugated to fluorescein (FITC). BrdU incorporation and PAcP expression were indicated using fluorescence microscopy. Histogram bars were created by counting a total of 100 cells from three different randomly chosen microscope fields. The number of cells expressing BrdU alone, BrdU and PAcP and PAcP alone are presented by the red, the yellow and the green bars, respectively. Data shown are mean ± S.D. (*n*=3). (**B**) **Differential localization of signal sequence chimera in LNCaP transfected cells.** Cells transfected as previously described were harvested at 72 h and analyzed for the presence of PAcP in cell lysate (top panel) and media (bottom panel) by WB after 10% SDS/PAGE. Equal amount of total protein was loaded on each lane. Data shown are % of control. Mean ± S.D. (*n*=12); *P<0.01, significantly different from control (wt-PAcP expression level). (**C**) **In gel acid phosphatase activity of putative PAcP forms.** Cells transfected with indicated constructs were harvested as previously, lysed and equal amount of cell lysate (left) and media (right) were separated by non-denaturing NGE, and acid phosphatase activity was measured in the gel using ELF 97 fluorogenic phosphate substrate in the absence (top gel panel) and presence (bottom gel panel) of 10 mM L(+)-tartrate as described in ‘Methods’ section. HSP was used as a positive control. Graph shows relative density of PAcP acid phosphatase activity. Data are mean ± S.D. (*n*=4); *P<0.03, significantly different from control (wtPAcP). Abbreviations: HSP, human seminal plasma; NGE, native gel electrophoresis; SDS/PAGE, polyacrylamide gel electrophoresis in sodium dodecyl sulfate.

To confirm these conclusions, transfected cells were labeled with bromodeoxyuridine (BrdU), whose incorporation serves as a measure of DNA synthesis [30]. Immunocytochemistry (ICC) was carried out using antibodies specific to BrdU and PAcP, to compare the distribution of PAcP expression versus cellular proliferation. Cells expressing IgG(NH)-PAcP do not incorporate BrdU, as shown by separate red and green cells in the field shown, and by quantitation of cells in multiple fields (Figure 3A). Thus, only the few cells *not* expressing PAcP are proliferating. In contrast, complete concordance of PAcP expression and BrdU incorporation is observed for Prl(NH)-PAcP transfected cells (all PAcP expressing cells are proliferating). Wt PAcP displays an intermediate effect. These results are consistent with the hypothesis that IgG(NH)PAcP favors expression of a form of PAcP (^GR^PAcP) responsible for androgen-sensitive growth regulation, and that Prl(NH)PAcP favors expression of another form (^PL^PAcP) responsible for androgen-independent growth.

### Distinctive subcellular localization of ^GR^PAcP and ^PL^PAcP

The ability of medium from Prl(NH)-PAcP to act as a growth factor for growth-arrested IgG(NH)-PAcP-expressing cells suggested a distinctive subcellular localization for this form of PAcP. This suspicion was confirmed when cell lysates and medium from transfected cells were analyzed by polyacrylamide gel electrophoresis in sodium dodecyl sulfate (SDS/PAGE) and WB, revealing a major difference in PAcP’s steady-state subcellular localization depending on which signal sequence chimera was being expressed (Figure 3B). Whereas the putative ^GR^PAcP expressed by IgG(NH)-PAcP transfected cells was almost entirely localized to an intracellular compartment, putative ^PL^PAcP expressed by Prl(NH)-PAcP transfected cells was efficiently secreted into the medium. In contrast, wtPAcP was observed to have predominantly intracellular PAcP at steady state, but with substantial quantities of a secretory form, consistent with greater heterogeneity than observed with the chimeric swapped signal sequences. That all forms of PAcP had entered the secretory pathway was confirmed by the presence of N-linked carbohydrates (shift with Endoglycosidase F [Endo F] digestion, data not shown). The significant differences in steady-state distribution between intracellular and secreted forms, achieved simply by swapping the N and H signal sequence domains, were corroborated by pulse-chase radiolabeling (data not shown). We next wished to determine whether the different functions associated with PAcP expression behind different signal sequences, correspond to differences in PAcP structure.

### Differences in PAcP function correlate with differences in PAcP structure

The different forms of PAcP were assayed for acid phosphatase activity for which the protein is best known. The in-gel phosphatase activity of PAcP made behind each signal sequence chimera was analyzed in cell lysates and found to be roughly equivalent (Figure 3C upper left hand panel). Medium from those cells (Figure 3C upper right hand panel) confirmed the subcellular localization differences noted previously. Then samples of cell lysate and medium were treated with L(+)-tartrate, and a distinctive L(+)-tartrate sensitivity profile was observed (Figure 3C lower gel panels) and quantified (Figure 3C, bar graphs below). IgG(NH)-PAcP transfected cells displayed complete inhibition of acid phosphatase activity by L(+)-tartrate, as classically described for PAcP [31]. In contrast, PAcP expressed in both cells and medium upon Prl(NH)PAcP transfection, was mainly L(+)-tartrate resistant. Wt-PAcP transfected cells displayed an intermediate degree of tartrate sensitivity in both cells and medium, consistent with a combination of L(+)-tartrate-sensitive and tartrate-resistant forms. PAcP from human seminal plasma (HSP) was observed to be exquisitely L(+)- tartrate sensitive, suggesting that this property is shared by at least two forms of PAcP, one that is retained in cells (putative ^GR^PAcP, magnified by IgG(NH)-PAcP expression), and another that is secreted into seminal fluid. Taken together, these studies demonstrate PAcP expression is functionally heterogeneous and that signal sequence swapping magnifies subsets of this heterogeneity, which correlates with differences in subcellular location.

### Structural studies of ^GR^PAcP, ^PL^PAcP, and ^SF^PAcP

We next established that the different forms of PAcP were identical in mature amino acid sequence. PAcP was purified by tartrate affinity chromatography from cell lysate of IgG(NH)-PAcP transfected cells (enriched in putative ^GR^PAcP), medium of Prl(NH)-PAcP transfected cells (enriched in putative ^PL^PAcP), and HSP (enriched in putative ^SF^PAcP) [31,32]. The authentic PAcP sequence in each case were identified (Supplementary Figure S6A). The fidelity of signal sequence: cleavage KELKFVTLVFRHG (D) was established by tandem mass spectrometry (Supplementary Figure S6B).

**Figure 4:**
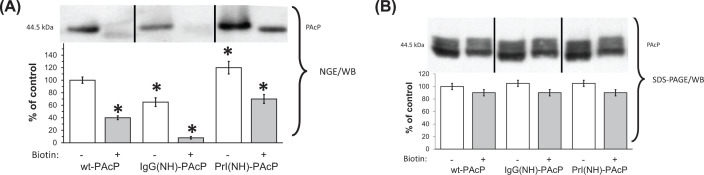
PAcP immunoreactivity before and after biotinylation (**A**) **NGE**. Cells were transfected as previously, harvested at 72 h, lysed and 20 µg of total protein used for the Sulfo-NHS-CC-Biotin coupling reaction (see ‘Methods’ section). NGE was conducted, followed by WB. (**B**) **SDS/PAGE.** Biotinylated lysates were treated by loading buffer for SDS/PAGE. The SDS/PAGE was conducted, followed by WB. White bars are without biotinylation, black bars with biotinylation. For quantitation, signal density was normalized to that of non- biotinylated wt-PAcP. Data shown are mean ± S.D. (*n*=4); *P<0.03, significantly different from control.

With primary amino acid sequence identity of the three putative functional forms of PAcP established, we characterized their non-covalent structural differences by various methods. Transfected cell lysates were analyzed by native gel electrophoresis (NGE) and WB (Figure 4 upper panel, biotin lanes) compared with SDS/PAGE and WB (Figure 4 lower panel, biotin lanes). Under native conditions, ^GR^PAcP was found to be significantly less immunoreactive to a PAcP antiserum compared with native putative ^PL^PAcP (Figure 4, lower panel, biotin lanes). Since chemical modification with biotin is known to markedly diminish mobility of peptide segments within a protein as examined by NMR [33], the samples were biotinylated under native conditions and the effect on immunoreactivity of the putative PAcP forms determined. Upon analysis by NGE and WB, a dramatic enhancement of the immunospecificity to ^PL^PAcP was observed. Note the change in ratio between the extreme right and middle white (− biotin) and black (+ biotin) bars in the upper panel of Figure 4, from approximately 2:1 to >8:1, respectively. Parallel samples subjected to NGE were subsequently analyzed by SDS/PAGE and WB to demonstrate equal presence of the protein in all lanes (data not shown), eliminating the trivial explanation that one form of PAcP failed to migrate on NGE or that biotinylation preferentially affected its ability to enter the gel. Upon analysis by SDS/PAGE and WB, all samples were found to be equally well biotinylated, with no significant differences in immunoreactivity after SDS/PAGE among the samples (Figure 4 lower panel). Note the slight shift to higher mw with biotinylation in all cases. These results suggest that biotinylation decreases ^GR^PAcP flexiblity and therefore maintains a critical immunoreactive epitope in a buried site. In contrast, ^PL^PAcP fully exposes that epitope on the cell surface and therefore remains strongly immunoreactive regardless of biotinylation.

We applied a tryptic digestion to determine possible structural difference between different transfectants. IgG(NH)-PAcP was found to be more resistant at 60 min of digestion, with no accumulation of digestion fragments (pattern 1, data not shown). Together these properties constitute a putative biochemical signature of ^GR^PAcP. Digestion of Prl(NH)-PAcP demonstrated two fragments (termed pattern 2), and no resistance at 60 min of digestion, a putative biochemical signature of ^PL^PAcP. In contrast, wtPAcP showed the digestion resistance signature of ^GR^PAcP, but a different digestion pattern than that of Prl(NH)-PAcP (termed pattern 2). These results suggested that ^GR^PAcP, ^PL^PAcP, and ^SF^PAcP were characterized by distinctive tryptic digestion patterns (data not shown), and predicted that PAcP in medium from ^wt^PAcP transfected cells was not ^PL^PAcP. This conclusion was also supported by the different cell sensitivity to PAcP in the medium from transfected cells (IgG(NH)-PAcP showed no effect on the growth rate), and by L(+)-tartrate affinity chromatography (see Supplementary Figure S7A,B).

The data from NGE immunoreactivity strongly support the notion that the identical mature PAcP sequence (as demonstrated by mass spectrometry), was folded differently to generate ^GR^PAcP and ^PL^PAcP. The data from acid phosphatase activity, L(+)-tartrate affinity chromatography, native tryptic mapping, and subcellular localization are all consistent with this conclusion. However these methods left some ambiguities: two forms are secreted (^PL^PAcP and ^SF^PAcP), and two forms (^GR^PAcP and ^SF^PAcP), have acid phosphatase activity that is L(+)-tartrate sensitive. Another structural method was needed to unambiguously distinguish these forms.

**Figure 5:**
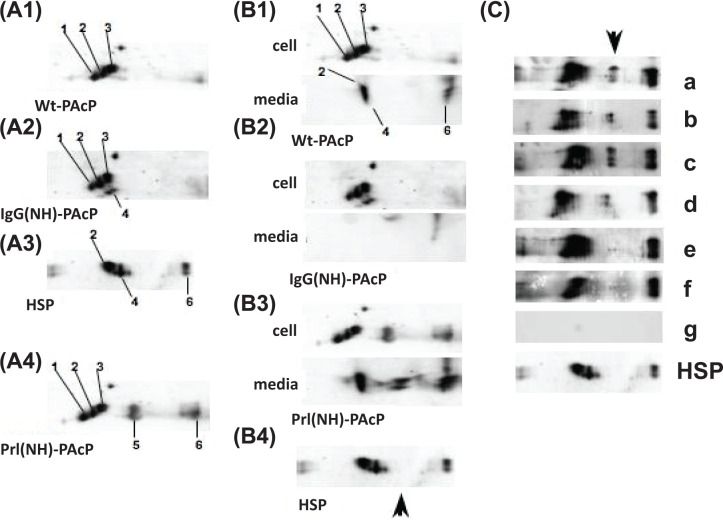
Analysis of PAcP forms by 2D IEF SDS/PAGE/WB Cells were transfected as previously, harvested at 72 h, lysed and 150 µg of total protein (lysate) or the same percentage of total medium was treated with Endo F to completion under native conditions, and analyzed using 2DGE followed by WB. (**A1**–**A4**) Analysis of transfected cell lysates was used to define six PAcP-related spots (see text). Wt-PAcP (A1), IgG(NH)-PAcP (A2), HSP (A3), Prl(NH)-PAcP (A4). (**B1**–**B4**) The spots defined from transfected cell lysates were compared with spots observed in HSP or media. Wt-PAcP (B1), IgG(NH)-PAcP (B2), Prl(NH)-PAcP (B3), HSP (B4), (**C**) 25 µl of human serum samples and 5 µl of HSP were precleaned, treated with Endo F to completion under native conditions, and analyzed by 2DGE as previously. Clinical histories were (a) Gleason grade 3+3, stage pT2cNXMX, adenocarcinoma; (b) Gleason grade 3+3, stage pT2cNXMX, small acinar adenocarcinoma; (c) Gleason grade 3+3, stage pT2cNXMX, small acinar adenocarcinoma; (d) Gleason grade 5+4, stage pT3N1MX, invasive prostatic adenocarcinoma; (e) Gleason grade 4+5, stage pT3bN1MX, small acinar. (f) Gleason grade 4+5, stage pT3bN1MX, small acinar; (g) normal serum. adenocarcinoma; (f) Gleason grade 4+5, stage pT2cN0MX, small acinar adenocarcinoma; (g) healthy male control serum; HSP as used in previous panels as a reference. See Supplementary Methods for additional details.

N-linked sugars were removed from all samples by exhaustive digestion with Endo F, and another analytical method, 2-dimensional gel electrophoresis (2DGE) involving isoelectric focusing (IEF) from pH 3 to 10 (first dimension), followed by SDS/PAGE (second dimension), was applied. A distinctive pattern of three groups of spots (#1–3) was observed in IgG(NH)PAcP transfected cells (Figure 5A second panel). An overlapping pattern of two spots (#2, #4), and another spot (#6) was observed in HSP (Figure 5A third panel). In addition to spots #1–3 and #6 in cell lysate, and #2, #4, and #6 in media (Figure 5B), a striking new spot (#5) was observed in Prl(NH)PAcP transfected cells in both cells and media (Figure 5A bottom panel). The 2DGE patterns after Endo F digestion provides further support for the hypothesis of subsets of PAcP involved in androgen-sensitive growth control (^GR^PAcP, spots #1–3), androgen-independent proliferation (^PL^PAcP, spot #5), and in HSP (^SF^PAcP, spot #2, #4, #6). Furthermore, these data suggest that wtPAcP comprises ^GR^PAcP (within cells) and ^SF^PAcP (secreted into the medium) but *not* ^PL^PAcP, and thus the non-L(+)-tartrate bound material expressed from wtPAcP transfected cells (data not shown) must represent yet other forms presently uncharacterized. One prediction of this analysis is that the medium from wtPAcP transfected cells contains ^SF^PAcP, not ^PL^PAcP, and hence, should *not* promote proliferation of growth-arrested IgG(NH)PAcP transfected cells. This prediction was fulfilled, validating the present approach and conclusions (Supplementary Figure S8).

With three forms of PAcP characterized and previous ambiguities of secretion and tartrate sensitivity resolved, we next explored a possible clinical correlation to prostate cancer. A set of blinded clinical serum samples were analyzed by 2DGE after Endo F digestion to remove sugars. When the code was subsequently broken, 2D gel spot #5, the signature of of Prl(NH)-PAcP transfected cells and medium, was observed in all of the early prostate cancer (Gleason stage 3+3) specimens (Figure 5C panels a–c). In contrast, normal serum showed no PAcP (Figure 5C panel g) and the late prostate cancers (Gleason stages 4+5 and 5+4, Figure 5C panels d–f) showed less or none of the ^PL^PAcP-specific spot #5. Consistent with our working hypothesis, the HSP PAcP 2D gel pattern was distinctly different from both ^GR^PAcP and ^PL^PAcP. Thus, ^SF^PAcP is likely a form of PAcP derived from yet another biosynthetic pathway. In light of the lack of growth factor activity of the L(+)-tartrate-resistant fraction of secreted PAcP expressed from wtPAcP transfected cells (Supplementary Figure S8), further heterogeneity of PAcP beyond these three forms cannot be excluded.

## Discussion

We demonstrate that simply swapping the N and H domains of the signal sequence has profound effects on function, subcellular localization, and structure of PAcP. These effects occur *without* differences in mature PAcP primary structure (Supplementary Figure S6). A number of lines of evidence, most notably Figure 4, suggest that differences in folding are critical determinants of the functional subsets of PAcP defined here, and hence that these forms can be considered as alternate conformers, generated by distinctive pathways of biogenesis, analogous to previous studies on the biogenesis of PrP and other proteins [10–15].

The different forms of PAcP also differently affected the classical oncogenic regulatory mechanism in LNCap cells: cyclinD/cdk4 complex. Cyclin D has been identified as a key regulatory subunit of the holoenzyme that promotes the G_1_/S-phase transition through phosphorylating the pRB protein [34]. Cyclin D1 overexpression leads to elevated cancer growth and negatively affects functions of proteins involved in DNA repair including BRCA1/2 [35,36]. Thus, the use of cyclin D-dependent kinase inhibitors was one of the approaches taken for the development of novel anticancer therapies, culminating in the development of palbociclib, which is being used effectively in the clinic for treatment of breast cancer [37]. Cyclin D1/2 and the kinases it regulates (cdk4 and cdk6) mediate the cellular response to mitogenic signals in androgen-induced prostate cell proliferation.

The changes in the abundance of cyclin D1/2 and cdk4/6 complexes and the phosphorylation state of Rb correlated with the percentage of proliferating cells before and after androgen treatment. Therefore, the overexpression of both complexes is a significant indicator for progression of the malignancy [38].

Conversely, if the cyclin D and cdk4/6 levels are decreasing, it shows that the prostate cancer cell phenotype is going back to less malignant one. This phenomenon, also correlated with restoring of the androgen sensitivity [39]. For example, Benitez et al., showed that expression of cyclin D1 and Cdk4 as well as cyclin D1/Cdk4 kinase activity were reduced under anticancer treatment in LNCaP, low passage (androgen-sensitive cancer line) and not in PC-3 (non-androgen sensitive cells) [40].

The androgen sensitivity of prostate cancer cells is one of the most important factors for the progression, treatment, and prognosis of the disease [41]. If they still have a prostate epithelium phenotype and have not lost the differentiation complexity of mature cells, they can be sensitive for the androgen blocking therapy [42]. However, if the cells become insensitive to the androgen/androgen blockers, the success of the anti-prostate cancer treatment might be very low. Thus, restoring of the androgen sensitivity is so important. The LNCaP cell line, developed by Horoszewicz, shows androgen-dependent growth in the early passages and a loss of androgen sensitivity in later stages [24,43]. It is important to mention that androgen treatment of these cells enhances the synthesis and secretion of PAcP [44].

LNCaP cells contain considerable amounts of androgen receptors [45]. We observed that effects of ^PL^PAcP are pro-oncogenic and correlated with the lost of the androgen sensitivity. Remarkably, ^GR^PAcP was able to restore the androgen sensitivity to cells and actually mimicked the effect of the androgen blocker in the experimental system.

**Figure 6:**
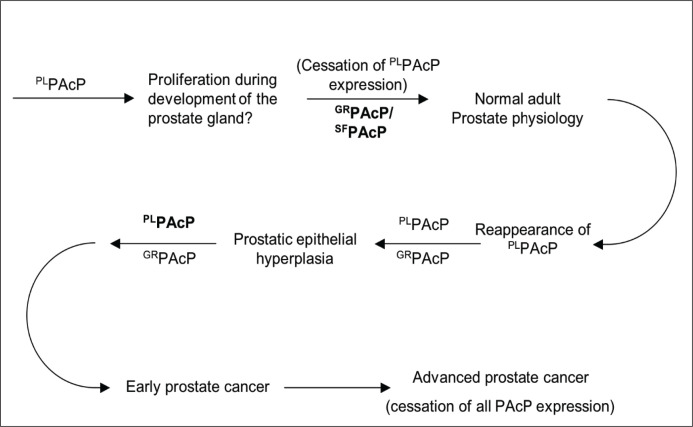
Proposed role of PAcP conformers in the natural history of prostate cancer See text for details.

In light of its demonstrated growth factor activity, and striking appearance in serum of patients with early prostate cancer, a role in prostate cancer pathogenesis is suggested for ^PL^PAcP (Figure 6). Given the evidence presented that ^PL^PAcP represents the product of a distinct pathway of biogenesis, its role in cancer likely represents dysregulated timing of expression rather than an aberration of structure *per se*. We therefore propose that ^PL^PAcP has a physiological function, perhaps of stimulating prostatic epithelial proliferation early in embryonic prostate development, but that its expression is normally developmentally programmed to be extinguished by adulthood. Dysregulated renewal of ^PL^PAcP expression in adulthood would provide an added stimulus to prostatic epithelial proliferation. As a result of its action, either malignant transformation is facilitated or incipient cancer progresses. Eventually, a high grade, androgen-independent malignant clone emerges in which PAcP is no longer expressed.

The striking lack of ^PL^PAcP within the heterogeneity represented by wtPAcP transfected cell lysate and medium (see Supplementary Figure S8), suggests that ^GR^PAcP and ^SF^PAcP are made by default pathways present in the LNCaP C-81 cell line that nolonger expresses PAcP. This is consistent with ^PL^PAcP being made via a tightly regulated pathway of biogenesis whose normal induction is temporally highly restricted. This reasoning suggests two novel anticancer therapeutic strategies. First, redirection of PAcP nascent chains from the induced pathway of ^PL^PAcP biogenesis to the likely cancer-suppressing default pathway of ^GR^PAcP biogenesis. Second, conformer-specific blockade of ^PL^PAcP growth factor action, to remove a critical early stimulus to cancer or its progression.

The present study also demonstrate that signal sequence swapping is a powerful tool for analysis of gene expression. It provides a means of managing the daunting heterogeneity of the proteome, identifying minor subsets of chains that may be critical to disease pathogenesis. A caveat is that the extent to which a given signal sequence will enrich for a particular conformer may need to be determined empirically for each protein studied. The differences in the behavior of different forms of PAcP are summarized in the Supplementary Table S1.

While the data presented provide striking evidence for conformational differences between subsets of PAcP structure that derive from distinct pathways of biogenesis, several areas of uncertainty remain. Exhaustive digestion with Endo F eliminated sugars as the sole basis for the differences observed (see Figure 5). However, glycosylation or other PTMs may still be *markers* of such differences. Likewise additional conformers of PAcP may have been missed by the present analysis, either due to the existence of other pathways of biogenesis discoverable by extension of signal sequence swapping methodology, or by other mechanisms.

Finally, these studies have a profound implication for our understanding of biological regulation of gene expression. Conventional proteomic analyses may miss critical features of proteins (e.g. protein folding and secondary covalent modifications) crucial to understanding normal physiology and natural history of disease. This has been shown to be the case for prion disease previously [46], has been implied for other proteins [47], and is extended here to PAcP and its role in prostate cancer.

Conversely, exhaustive analysis of the proteome generates an enormous ‘haystack’ of heterogeneity in which the pathophysiologically relevant ‘needle’ is difficult to determine. Here we used distinctive pathways of biogenesis to filter this heterogeneity and thereby identify those forms of particular interest. The robust functional differences and correlation to prostate cancer elucidated, suggests this strategy can be powerful.

The occurrence of alternate forms of proteins with radically different functions suggests a great deal more information content in the genome than is generally appreciated. A critical challenge of the coming period is to learn how to detect, organize, and manipulate this functional conformational diversity in a rapid and facile manner. The tools used here can help, but point to a more daunting future challenge: the task of dissecting the transient, co-translational pathways by which this new level of biological regulation occurs.

## Methods

### LNCaP cell model and cell culture

The LNCaP cell model is a well accepted prostate cancer cell line (American Type Culture Collection, Manassas, Virginia, United States). Briefly, we used two stages of LNCaP cells during regular maintenance designated as C33 (LNCaP passage number <33) and C-81 (LNCaP passage number between 81 and 120). The cells in these stages exhibit different androgen-responsiveness of growth stimulation: C33 cells are androgen-sensitive while C-81 cells are androgen independent. This cell model recapitulates the progression of human prostate tumor from early to the advanced hormone-refractory stage. Cells were routinely maintained in RPMI 1640 medium supplemented with 5% FCS, 1% L-glutamine, 50 µg/ml gentamycin sulfate. All tissue culture material was from GIBCO.

### Generation of constructs

All constructs were engineered using standard PCR and cloning techniques. PCRs were performed with a universal reverse primer designed complementary to the 3′ nucleotide sequence of PAcP and long forward primers (ranging from 80 to 120 base pairs) harboring the appropriate nucleotides encoding various signal sequences. To eliminate undesirable mutations associated with long PCRs, only a small fragment of the PCR product was subcloned into pBluescript II SK vector using a unique HincII restriction site in PAcP sequence at base pair 327. Clones were then sequenced to ensure successful generation of nucleotide swap without unwanted mutations. PAcP variants were then transferred from pBluescript II SK vector to either pTNT or pcDNA3.1/Zeo (+) vectors by restriction digestion and ligation.

### Androgen responsiveness assay

For transient transfection experiments, cells were plated at a density of 4 × 10^5^ cells per well in a six-well plate in RPMI 1640 medium containing 5% charcoal/dextran-stripped FBS (cFBS) 24 h before transfection. The adherent cells were transfected using 10 µl cationic lipid reagent Lipofectamine 2000 (Invitrogen) with 4 µg of plasmid DNA for PAcP in the serum-free RPMI 1640 medium. After 5 h of incubation, transfection medium was replaced with 2 ml/well complete medium supplement with cFBS. Seventy-two hours after transfection, cells were trypsinized and counted using counting chamber (hemocytometer). For the androgen sensitivity growth control experiments, 10 nM of 5α-dihydrotestosterone (DHT) (Sigma) was added at last 48 h of transfection.

### Generation of stable cell lines

LNCaP clone C-81 cells were transfected with pcDNA3.1zeo+ vector harboring full-length human PAcP with IgG(NH)-PAcP or Prl(NH)-PAcP signal sequences. Stable cell lines were selected under 500 µg of zeocin (Invitrogen). Expression of PAcP was confirmed by Western immunoblotting and ICC staining using anti-PAcP antibody (see below).

### Growth assay in the presence of Prl(NH)-PAcP transfected media

C-81 cells were transfected as described above with pcDNA3.1/Zeo(+) vector harboring full-length human Prl(NH)-PAcP or empty vector (mock). Seventy-two hours after transfection medium was harvested. The Prl(NH)-PAcP medium or empty vector transfected cells medium was introduced instead of the growing medium to the wt-PAcP, IgG(NH)-PAcP, Prl(NH)-PAcP and mock transfected cells after 5 h of experimental transfection (2 ml/well). Seventy-two hours later, all types of cells were trypsinized and counted by hemocytometer. For these experiments acid phosphatase enzymatic activity of Prl(NH)-PAcP media was established. Acid phosphatase activity was assayed in a reaction mixture (0.1 ml) containing 10 mM *p*-nitrophenyl phosphate (pNPP; Sigma, St. Louis, Missouri, U.S.A.), 100 mM sodium acetate, pH 5.0, at room temperature. The assay was initiated with addition of 5 µl medium containing Prl(NH)-PAcP enzyme or mock-transfected medium. Appropriate controls in which non-transfected complete media were included with each experiment to correct the absorbance due to the color of the complete 1640 RPMI media (with Phenol Red) containing 5% cFBS. Non-enzymatic hydrolysis of pNPP was corrected by including control assays without added enzyme. The amount of product, *p*-nitrophenol, produced was calculated from the increase in absorbance at 410 nm using the molar extinction coefficient of 17800 M^−1^.cm^−1^, which was determined with *p*-nitrophenol standards.

One unit of enzyme is defined as the amount of enzyme that is required to hydrolyze 1 pmol of pNPP/min at room temperature. Western immunoblotting with anti-PAcP antibody (10 µl medium) was used as additional control for Prl(NH)-PAcP expression.

### Western immunoblotting

C-81 cells were transfected as describe above. Seventy-two hours after transfection, cells were washed with ice-cold PBS and lysed into 150 mM HEPES, 50 mM NaCl, 0.1% Triton-X 100, 2 mM PMSF, and 1:100 (v/v) protease inhibitors cocktail (Sigma). The protein concentration of cell extracts was measured by Bradford’s method [48] using the Bio-Rad protein assay kit (Bio-Rad Laboratories) with bovine serum albumin as a standard and 10–20 µg of total protein or relative amount of media for each transfection was separated at 10% SDS/PAGE or non-denatured PAGE, followed by WB with anti-PAcP antibody raised against purified denatured PAcP from HSP.

Mouse mAb against tubulin was used as a loading control. WB were developed and band intensities were measured using Typhoon (GE Healthcare, Buffalo, N.Y.) imaging system or band intensities were quantified with NIH Image software.

### Western immunoblotting for phosphorylated proteins

LNCaP C-81 transfected cells were washed three times with ice-cold PBS and lysed into NaCl 150 mM, Tris pH 7.5 50 mM, NP-40 1% (v/v), EDTA 1 mM, EGTA 1 mM, Na_3_VO_4_ 1 mM, NaF 50 mM, Na_4_P_2_O_7_ 10 mM, Na deoxycholate 0.25% (v/v), Na β-glycerophosphate 10 mM, β-mercaptoethanol 1% (v/v), PMSF 1 mM, protease inhibitors coctail 1:100 (v/v). The protein concentration of cell extracts was measured by Bradford’s method and amount of 20–60 µg of total protein for each individual transfection was separated at 6–12% SDS/PAGE, followed by Western immunoblotting with anti-pY clone 4G10 (Upstate), anti-CyclinD 1/2 (Upstate), anti-Cdk 4 (Santa Cruz), anti-phosphoTyr^317^ Shc p52 (Upstate) antibodies. A mouse mAb against tubulin was used a loading control. WB were developed and band intensities were measured using Typhoon Imaging system.

### Affinity chromatography of PAcP

L(+)-Tartrate (Fisher Scientific, New Jersey, U.S.A.) was coupled to column chromatography media as described [49] and equilibrated with sodium acetate buffer (50 mmol/liter, pH 5.0). For mass spectrometry analysis of PAcP from Prl(NH)-PAcP transfected media, IgG(NH)-PAcP cell lysate, or HSP, the protein was eluted with 50 mmol/l L(+)-tartrate in sodium acetate buffer. For affinity chromatography gradient analysis, cell lysates and HSP were applied on to the column (176 µg of total protein and 5 µl HSP, respectively). The level of PAcP was previously normalized by Western blot.

The enzyme was then eluted by linear gradient of the same buffer containing from 0 to 10 mM L(+)-tartrate with a flow rate of 26 ml/h. Fractions were dialyzed against Tris-buffered saline overnight, lyophilized and stored at −80°C. The affinity chromatography gradient samples were resuspended in Laemmli sample buffer and one-fifth of each fraction applied to 10% SDS/PAGE and analyzed by WB using anti-PAcP antibody. The column was reused after three cycles of washing in 0.5 M sodium chloride in 0.1 M sodium acetate buffer pH 4.0; 0.5 M sodium chloride in 0.1 M sodium bicarbonate buffer pH 9.0 in water.

### ICC for BrdU incorporation and PAcP expression

LNCaP clone C-81 cells were plated in six-well plates on the slide at a density of 10^5^ cells/slide and were transfected with pcDNA3.1zeo+ vector harboring full-length human PAcP with different signal sequences. Forty-eight hours after transfection cells were incubated with 10 µM 5-bromo-2-deoxyuridine (BrdU) (Sigma) at 37°C for 1 h, washed and fixed with 95% ethanol and 5% acetic acid. DNA was denatured by incubation in 2 N HCl/0.5% Triton X-100 buffer. After the cells were washed, blocked with 5% BSA and stained with anti-BrdU [2B1] mAb (Abcam) followed by secondary anti-mouse antibody conugated to cyanine CY3 (Jackson Immuno Research). Then cells were incubated with polyclonal anti-PAcP antibody followed by incubation with secondary anti-rabbit antibody conugated to fluorescein (FITC) (Jackson Immuno Research). BrdU incorporation and PAcP expression were indicated using fluorescence microscopy.

### In gel acid phosphatase activity assay

C-81 transfected cells were harvested, lysed, and relatively equal amounts of cell lysate and media were separated on non-denatured PAGE. In gel acid phospatase activity was detected as followed: gels were incubated in 100 mM sodium acetate buffer pH 5.0 in the presence or absence of 10 mM L(+)-tartrate for 30 min, then gels were briefly washed with the buffer without L(+)-tartrate and replaced with the same buffer contain 5 µM Enzyme-Labeled-Fluorescence (ELF 97) (Molecular Probes) fluorogenicphosphate substrate for 5 min. The bands were visualized under UV excitation.

### Tryptic mapping of native PAcP forms

Twenty micrograms of total cellular protein from standard cell transfection was incubated at 37°C for 15 and 60 min with final concentration of trypsin 20 or 100 µg/ml in 40 mM Tris (pH 8.1), 1 mM CaCl_2_ digestion buffer. Reactions were stopped by addition of PMSF to 0.5 mM, EDTA to 10 mM and aprotinin to 0.03 mg/ml. After incubation for 10 min, samples were prepared in Laemmli sample buffer and resolved to 12% SDS/PAGE. and analyzed by WB using anti-PAcP antibody. Control treatments were carried under identical conditions without adding trypsin.

### Biotinylation of lysine residues on PAcP forms

Twenty micrograms of total protein was used for the Sulfo-NHS-CCBiotin (Pierce) coupling reaction. Biotinylation was done in PBS for 23 min at 28°C at a final concentration of 10 mM biotin. The reaction was stopped by adding excess (final concentration 20 mM) free lysine. Control treatments were carried out under identical conditions without biotin.

### Sample preparation for mass spectrometry

To establish that the proteins were identical in amino acid sequence by mass determination and tandem mass spectrometry of the amino terminal peptides the intracellular IgG(NH)-PAcP, secreted Prl(NH)-PAcP from the media and PAcP from seminal fluid were first purified on L(+)-tartrate column (as described), then purified PAcP (40 µg) was dissolved in 20 µl of 6 M guanidinium hydrochloride, 0.5 M sodium phosphate (pH 8.0), 0.125 M dithiothreitol (DTT) and incubated at 60°C for 1 h. After cooling to room temperature, 10 µl of 1 M iodoacetamide was added and the mixture incubated in the dark for 1.5 h. The sample volume was increased to 300 µl with 0.1 M sodium phosphate (pH 8.0). The pH was adjusted to ∼7.5 by addition of 15 µl of 0.5 M sodium phosphate (pH 8.0). Endoproteinase Asp-N (0.6 mg) (sequencing grade Endoproteinase Asp-N was obtained from Wako Chemicals U.S.A., Inc. (Richmond, VA)) was added and the mixture was incubated at 37°C in the dark for 36 h. Digestion was terminated by addition of trifluoracetic acid and the samples were stored at −80°C until analysis by mass spectrometry.

### Liquid chromatography tandem mass spectrometry

Mixtures of proteolytically generated peptides were analyzed by nano-liquid chromatography tandem mass spectrometry (LC-MS/MS) utilizing an Eksigent 2DLC nanoHPLC System (Eksigent, Dublin, CA) interfaced with a QStar XL mass spectrometer (Applied Biosystems/Sciex) equipped with an ionspray II nanospray source. External calibration was performed in MS/MS mode using fragment ions of Glu-fibrinopeptide as references. A Pepmap C18 trap column (300 µm i.d., 5 mm length, 300 Å pore size, 5 µm particle size, LC Packings, Sunnyvale, CA) and a 15 cm, 75 µm column self-packed with Jupiter Proteo C14 end-capped material (90Å pore size, 4 µm particle size, Phenomenex, Torrance, CA) were used for desalting and reversed phase peptide separation, respectively. A 25-min linear gradient from 2% B to 55% B was run at 250 nl/min flow rate, utilizing solvent A: 2% acetonitrile/0.1% formic acid and solvent B: 80% acetonitrile/0.08% formic acid. Precursor ion selection employed an automated routine (IDA, Analyst QS 1.1. Applied Biosystems/Sciex) that consisted of a series of one survey MS scan (1 s, m/z 400–1700) and two MS/MS scans (2 s, m/z 60–1500); nitrogen served as a collision gas and collision energy was automatically adjusted depending on the size and charge state of the precursor ion. The doubly and triply charged molecular ions representing peptides KELKFVTLVFRHG as well as a putative peptide DRSVLAKELKFVTLVFRHG, the latter expected in the presence of signal peptide sequence, were included into the IDA precursor ion selection program to preferentially perform MS/MS scans on any ions ±150 ppm of the input *m/z*. Protein identification was accomplished by using the MASCOT 2.1 (Matrix Science) search engine. Mammalia taxonomy was searched within the MSDB (08/31/2006) database utilizing the following parameters: precursor ion mass tolerance: 150 ppm; fragment mass tolerance: 0.15 Da; Asp-N digestion with three missed cleavages, fixed modifications: S-carboxyamidomethyl, variable modifications: deamidation (Asn and Gln); Met-sulfoxide; Pyro-Glu (from N-terminal Gln); phosphorylation of Ser and Thr. The analysis was performed at the Mass Spectrometric Facility of the University of Califonia San Francisco.

### 2DGE and WB

Prior to the 2DGE/WB analysis, 150 µg of total protein and 100 µl of media from each separate transfection were treated under native conditions with Endo F (New England BioLab) for 1 h at 37°C (amount of Endo F added to the reaction was calculated as 1 U/10 µg of total protein, prior WB was performed to assure that sugars removed completely). A total of 25 µl of human blood samples and 5 µl of HSP were precleaned with ProteoPrep Immunoaffinity Albumin&IgG Depletion Kit (Sigma) and treated with Endo F as described above. Each sample (cell lysis, media, blood sample, and seminal plasma) after Endo F digestion was individually dissolved in a rehydration buffer (8 M urea, 2% 3-[C3-cholamidoproyl] dimethyl-ammonio-1-propansulfonate [CHAPS], 50 mM DTT, 0.2% Bio-Lyte 3/10 ampholyte, 0.002% Bromophenol Blue), and subjected to 2D gel electrophoresis (IEF in the first dimension). Briefly, strips were incubated overnight with ReadyStrip IPG Strips (pH range of 3–10 NL) at room temperature. The IEF separation was carried out in PROTEAN IEF Cell by following the manufacturer’s suggested method (Bio-Rad Laboratories, Hercules, CA). Following the IEF separation, the strips were consecutively treated for 10 min each, first with 1% (w/v) DTT (in equilibration buffer: 0.375 M Tris, pH 8.8, containing 6 M urea, 20% glycerol, and 2% SDS), and next with 2.5% (w/v) iodoacetamide (also dissolved in equilibration buffer). IEF was performed at 20°C, with the following focusing parameters: 250 V (15 min), then from 250 to 4000 V with the 10000 V.hour. The current was limited to 50 µA/strip. After IEF, the individual strips were washed in SDS electrophoresis buffer (25 mM Tris pH8.8, 192 mM glycine, 0.1% SDS), placed on top of 10% SDS/PAGE and sealed in place with sealing solution (0.5% low-melting agarose in SDS electrophoresis buffer). The proteins were transferred to PVDF membrane and immunoblotted with anti-PAcP antibody.

### Statistical analysis

The results are given as mean ± SE. The statistical significance *(*P*<0.05) was calculated among the experimental groups using Whitney–Mann U-test. The QuickCalcs online service GraphPad Software, found at www.graphpad.com/quickcalcs/ttest1.cfm, was used for statistical evaluations.

## Supplementary Material

Supplementary Figures S1-S8 Table S1

## Data Availability

All generated data are presented in the article.

## Abbreviations

2DGE: 2-dimensional gel electrophoresis
BrdU: bromodeoxyuridine
cFBS: charcoal/dextran-stripped FBS
DTT: dithiothreitol
Endo F: endoglycosidase F
ER: endoplasmic reticulum
HSP: human seminal plasma
ICC: immunocytochemistry
IEF: isoelectric focusing
IgG: immunoglobulin G
mAb: monoclonal antibody
NGE: native gel electrophoresis
PAcP: prostatic acid phosphatase
Prl: prolactin
PrP: prion protein
PTM: post-translational modification
RMJ: ribosome–membrane junction
SDS/PAGE: polyacrylamide gel electrophoresis in sodium dodecyl sulfate
WB: Western blotting

## References

[1] Nelson W.G., De Marzo A.M. and Isaacs W.B. (2003) Prostate cancer. N. Engl. J. Med. 349, 366–381 10.1056/NEJMra021562 12878745

[2] Lin M.F., Meng T.C., Rao P.S., Chang C.S., Schonthal A.H. and Lin F.F. (1998) Expression of human prostatic acid phosphatase correlates with androgen- stimulated cell proliferation in prostate cancer cell lines. J. Biol. Chem. 273, 5939–5947 10.1074/jbc.273.10.5939 9488733

[3] Sinha A.A., Gleason D.F., Wilson M.J., Wick M.R., Reddy P.K. and Blackard C.E. (1998) Relationship of prostatic acid phosphatase localization in human prostate by a monoclonal antibody with the Gleason grading system. Prostate 13, 1–15 10.1002/pros.2990130102 2458582

[4] Meng T.C., Lee M.S. and Lin M.F. (2000) Interaction of protein tyrosine phosphatase and protein tyrosine kinase is involved in androgen-promoted growth of human prostate cancer cells. Oncogene 19, 2664–2677 10.1038/sj.onc.1203576 10851066

[5] Muniyan S., Chaturvedi N.K., Dwyer J.G., Lagrange C.A., Chaney W.G. and Lin M.F. (2013) Human prostatic acid phosphatase: structure, function and regulation. Int. J. Mol. Sci. 14, 10438–10464 10.3390/ijms140510438 23698773 PMC3676848

[6] Kong H.Y. and Byun J. (2013) Emerging roles of human prostatic acid phosphatase. Biomol Ther. 21, 10–20 10.4062/biomolther.2012.095 PMC3762301 24009853

[7] Veeramani S., Yuan T.C., Chen S.J., Lin F.F., Petersen J.E., Shaheduzzaman S. et al. (2005) Cellular prostatic acid phosphatase: a protein tyrosine phosphatase involved in androgen- independent proliferation of prostate cancer. Endocr. Relat. Cancer 12, 805–822 10.1677/erc.1.00950 16322323

[8] Tanaka M., Kishi Y., Takanezawa Y., Kakehi Y., Aoki J. and Arai H. (2004) Prostatic acid phosphatase degrades lysophosphatidic acid in seminal plasma. FEBS Lett. 571, 197–204 10.1016/j.febslet.2004.06.083 15280042

[9] Brillard-Bourdet M., Rehault S., Juliano L., Ferrer M., Moreau T. and Gauthier F., Amidolytic activity of prostatic acid phosphatase on human semenogelins and semenogelin-derived synthetic substrates. Eur. J. Biochem. 269, 390–395 10.1046/j.0014-2956.2001.02667.x 11784334

[10] Chu T.M. and Lin M.F. (1998) PSA and acid phosphatase in diagnosis of prostate cancer. J. Clin. Ligand Assay 21, 1–11

[11] Bedatty Fernandes F.C. and Bueno P.R. (2017) Optimized electrochemical biosensor for human prostatic acid phosphatase Sens. Actuat. B Chem. 253, 1106–1112

[12] Kong H.Y. and Byun J. (2015) Screening and characterization of a novel RNA aptamer that specifically binds to human prostatic acid phosphatase and human prostate cancer cells. Mol. Cell 38, 171–179 10.14348/molcells.2015.2272 25591398 PMC4332034

[13] Hegde R.S., Voigt S. and Lingappa V.R. (1998) Regulation of protein topology by trans-acting factors at the endoplasmic reticulum. Mol. Cell 2, 85–89 10.1016/S1097-2765(00)80116-1 9702194

[14] Rutkowski D.T., Lingappa V.R. and Hegde R.S. (2002) Substrate-specific regulation of the ribosome-translocon junction by N-terminal signal sequences. Proc. Natl. Acad. Sci. U.S.A. 98, 7823–7828 10.1073/pnas.141125098 PMC35426 11416167

[15] Rutkowski D.T., Ott C.M. and Lingappa V.R. (2003) Signal sequences initiate the pathway of maturation in the endoplasmic reticulum lumen. J. Biol. Chem. 278, 30365–30372 10.1074/jbc.M302117200 12771148

[16] Lingappa V.R., Rutkowski D.T., Hegde R.S. and Andersen O.S. (2002) Conformational control through translocational regulation: a new view of secretory and membrane protein folding. Bioessays 24, 741–748 10.1002/bies.10130 12210535

[17] Ghosh D.K. and Ranjan A. (2020) The metastable states of proteins. Protein Sci. 29, 1559–1568 10.1002/pro.3859 32223005 PMC7314396

[18] Blobel G. (2000) Protein targeting (Nobel lecture). ChemBioChem 1, 86–102 10.1002/1439-7633(20000818)1:2<86::AID-CBIC86>3.0.CO;2-A 11828402

[19] Fons R.D., Bogert B.A. and Hegde R.S. (2004) Substrate-specific function of the translocon-associated protein complex during translocation across the ER membrane. J. Cell Biol. 160, 529–539 10.1083/jcb.200210095 PMC2173754 12578908

[20] Kim S.J., Rahbar R. and Hegde R.S. (2001) Combinatorial control of prion protein biogenesis by the signal sequence and transmembrane domain. J. Biol. Chem. 276, 26132–26140 10.1074/jbc.M101638200 11359769

[21] Hegde R.S. and Lingappa V.R. (1996) Sequence-specific alteration of the ribosome-membrane junction exposes nascent secretory proteins to the cytosol. Cell 85, 217–228 10.1016/S0092-8674(00)81098-3 8612274

[22] Chuck S.L. and Lingappa V.R. (1992) Pause transfer: a topogenic sequence in apolipoprotein B mediates stopping and restarting of translocation. Cell 68, 9–15 10.1016/0092-8674(92)90202-N 1370657

[23] Tuteja R. (2005) Type I signal peptidase: an overview. Arch. Biochem. Biophys. 441, 107–111 10.1016/j.abb.2005.07.013 16126156

[24] Horoszewicz J.S., Leong S., Kawinski E., Karr J.P., Rosenthal H., Chu T.M. et al. (1983) LNCaP model of human prostatic carcinoma. Cancer Res. 43, 1809–1818 6831420

[25] Igawa T., Lin F.F., Lee M.S., Karan D., Batra S.K. and Lin M.F. (2002) Establishment and characterization of androgen-independent human prostate cancer LNCaP cell model. Prostate 50, 222–235 10.1002/pros.10054 11870800

[26] Culig Z. (2019) Epithelial mesenchymal transition and resistance in endocrine-related cancers. Rev. Biochim. Biophys. Acta. Mol. Cell Res. 1866, 1368–1375 10.1016/j.bbamcr.2019.05.003 31108117

[27] Yan S., Liu H., Liu Z., Peng F., Jiang F., Li L. et al. (2020) CCN1 stimulated the osteoblasts via PTEN/AKT/GSK3β/cyclinD1 signal pathway in Myeloma Bone Disease. Cancer Med. 9, 737–744 10.1002/cam4.2608 31769620 PMC6970049

[28] Crissman H.A. and Steinkamp J.A. (1990) Detection of bromodeoxyuridine-labeled cells by differential fluorescence analysis of DNA fluorochromes. Methods Cell Biol. 33, 199–206 10.1016/S0091-679X(08)60525-7 1707483

[29] Bates S., Parry D., Bonetta L., Vousden K., Dickson C. and Peters G. (1994) Absence of cyclin D/cdk complexes in cells lacking functional retinoblastoma protein. Oncogene 9, 1633–40 8183557

[30] Vega-Avila E. and Pugsley M.K. (2011) An overview of colorimetric assay methods used to assess survival or proliferation of mammalian cells. Proc. West. Pharmacol. Soc. 54, 10–14 22423572

[31] Vihko P., Kontturi M. and Korhonen K. (1978) Purification of human prostatic acid phosphatase by affinity chromatography and isoelectric focusing. Part I. Clin. Chem. 24, 466–470 10.1093/clinchem/24.3.466 630708

[32] Blass K.G. and Ho C.S. (1983) Electrochemical determination of orthophosphoric monoester phosphohydrolase activity (EC 3.1.3.1 and EC 3.1.3.2 alkaline and acid phosphatases, United States Patent, number 4406751. https://patents.justia.com/patent/4406751

[33] Yao X., Soden C., Summers M.F. and Beckett D. (1999) Comparison of the backbone dynamics of the apo- and holo-carboxy- terminal domain of the biotin carboxyl carrier subunit of Escherichia coli acetyl-CoA carboxylase. Protein Sci. 8, 307–317 10.1110/ps.8.2.307 10048324 PMC2144255

[34] Di Sante G., Di Rocco A., Pupo C., Casimiro M.C. and Pestell R.G. (2017) Hormone-induced DNA damage response and repair mediated by cyclin D1 in breast and prostate cancer. Oncotarget 8, 81803–81812 10.18632/oncotarget.19413 29137223 PMC5669849

[35] Wang C., Fan S., Li Z., Fu M., Rao M., Ma Y. et al. (2005) Cyclin D1 antagonizes BRCA1 repression of estrogen receptor alpha activity. Cancer Res. 65, 6557–6567 10.1158/0008-5472.CAN-05-0486 16061635

[36] Jirawatnotai S., Hu Y., Michowski W., Elias J.E., Becks L., Bienvenu F. et al. (2011) Nature 474, 230–234 10.1038/nature10155 21654808 PMC3134411

[37] Sherr C.J., Beach D. and Shapiro G.I. (2016) Targeting CDK4 and CDK6: from discovery to therapy. Cancer Discov. 6, 353–367 10.1158/2159-8290.CD-15-0894 26658964 PMC4821753

[38] Chen Y., Robles M.Y., Martinez L.A., Liu F., Gimenez-Conti I.B. and Conti C.J. (1996) Expression of GI cyclins, cyclin-dependent kinases, and cyclin-dependent kinase inhibitors in androgen-induced prostate proliferation in castrated rats. Cell Growth Differ. 7, 1571–1578 8930407

[39] Yao M., Xie C., Kiang M.Y., Teng Y., Harman D., Tiffen J. et al. (2015) Targeting of cytosolic phospholipase A2α impedes cell cycle re-entry of quiescent prostate cancer cells. Oncotarget 6, 26370–26386 10.18632/oncotarget.5277 PMC4741466 26416244

[40] Benitez D.A., Pozo-Guisado E., Alvarez-Barrientos A., Fernandez-Salguero P.M. and Castellón E.A. (2007) Mechanisms involved in resveratrol-induced apoptosis and cell cycle arrest in prostate cancer-derived cell lines. J. Androl. 28, 282–293 10.2164/jandrol.106.000968 17050787

[41] Koochekpour S. (2010) Androgen receptor signaling and mutations in prostate cancer. Asian J. Androl. 12, 639–657 10.1038/aja.2010.89 20711217 PMC3006239

[42] Vickman R.E., Franco O.E., Moline D.C., Vander Griend D.J., Thumbikat P. and Hayward S.W. (2020) The role of the androgen receptor in prostate development and benign prostatic hyperplasia: a review. Asian J Urol. 7, 191–202 10.1016/j.ajur.2019.10.003 32742923 PMC7385520

[43] Berns E.M., de Boer W. and Mulder E. (1996) Androgen dependent growth regulation and the release of specific protein(s) by the androgen receptor containing human prostate tumor cell line LNCaP. Prostate 9, 247–259 10.1002/pros.2990090305 2946029

[44] Schulz P., Bauer H.W. and Fittler F. (1985) Steroid hormone regulation of prostatic acid phosphatase expression in cultured human prostatic carcinoma cells. Biol. Chem. 366, 1033–1039 10.1515/bchm3.1985.366.2.1033 3878148

[45] Schuurmans A.L.G., Bolt J., Voorhorst M.M., Blankenstein R.A. and Mulder E. (1988) Regulation of growth and epidermal growth factor receptor levels of prostate tumor cells by different steroids. Int. J. Cancer 42, 917–922 10.1002/ijc.2910420622 3263955

[46] Hegde R.S., Mastrianni J.A., Scott M., DeFea K.D., Tremblay P., Torchia M. et al. (1998) Pathogenesis of spontaneous neurodegenerative disease involves a transmembrane form of the prion protein. Science 279, 827–834 10.1126/science.279.5352.827 9452375

[47] Hegde R.S., Trembley P., Groth D., Prusiner S.B. and Lingappa V.R. (1999) Transmissible and genetic prion diseases share a common pathway of neurodegeneration involving transmembrane prion protein. Nature 402, 822–826 10.1038/45574 10617204

[48] Bradford M.A. (1975) Rapid and sensitive method for the quantitation of microgram quantities of protein utilizing the principle of protein-dye binding. Anal. Biochem. 72, 248–254 10.1016/0003-2697(76)90527-3 942051

[49] Cuatrecasas P. (1970) Protein purification by affinity chromatography. Derivatizations of agarose and polyacrylamide beads. J. Biol. Chem. 245, 3059–3065 10.1016/S0021-9258(18)63022-4 5432796

[50] Ott C.M. and Lingappa V.R. (2004) Signal sequences influence membrane integration of the prion protein. Biochemistry 43, 11973–11982 10.1021/bi049156s 15379537

[51] Shields D. and Blobel G. (1978) Efficient cleavage and segregation of nascent presecretory proteins in a reticulocyte lysate supplemented with microsomal membranes. J. Biol. Chem. 253, 3753–3756 10.1016/S0021-9258(17)34748-8 649601

[52] Lingappa V.R., Lingappa J.R., Prasad R., Ebner K.E. and Blobel G. (1978) Coupled cell-free synthesis, segregation, and core glycosylation of a secretory protein. Proc. Natl. Acad. Sci. U.S.A. 75, 2338–2342 10.1073/pnas.75.5.2338 276877 PMC392548

[53] Skach W.R., Calayag M.C. and Lingappa V.R. (1993) Evidence for an alternate model of human P-glycoprotein structure and biogenesis. J. Biol. Chem. 268, 6903–6908 10.1016/S0021-9258(18)53125-2 8096508

