# Supplementary material for: Multifunctionality of prostatic acid phosphatase in prostate cancer pathogenesis
Source: Biosci Rep. 2021 Oct 22;41(10):BSR20211646. doi: 10.1042/BSR20211646 (PMC8536833; doi:10.1042/BSR20211646)
Supplement: Supplementary Figures S1-S8 Table S1 [file BSR-2021-1646_supp.pdf]

Supplementary Materials

Multifunctionality of Prostatic Acid Phosphatase in Prostate Cancer Pathogenesis

Evgenia Alpert[1], Armin Akhavan[1], Arie Gruzman[1], William J. Hansen[2] , Joshua Lehrer-Graiwer[2], Steven C. Hall[4,7], Eric Johansen[5,7], Sean McAllister[6], Mittul Gulati[3], Ming-Fong Lin[8] and Vishwanath R. Lingappa[1,2,3]

[1] Bioconformatics and [6]Cancer Laboratories of the California Pacific Medical Center (CPMC) Research Institute, 475 Brannan St, SF, CA 94107; [2]Prosetta Corporation, 670 5th St, SF, CA 94107; [3]Department of Physiology, [4]Cell and Tissue Biology, [7]Biomolecular Resource Center Mass Spectrometry Facility, and [5]Cancer Center, UCSF, 513 Parnassus Ave, SF, CA 94143; [8]Department of Biochemistry and Molecular Biology, University of Nebraska Medical Center, Omaha, NE 68198.

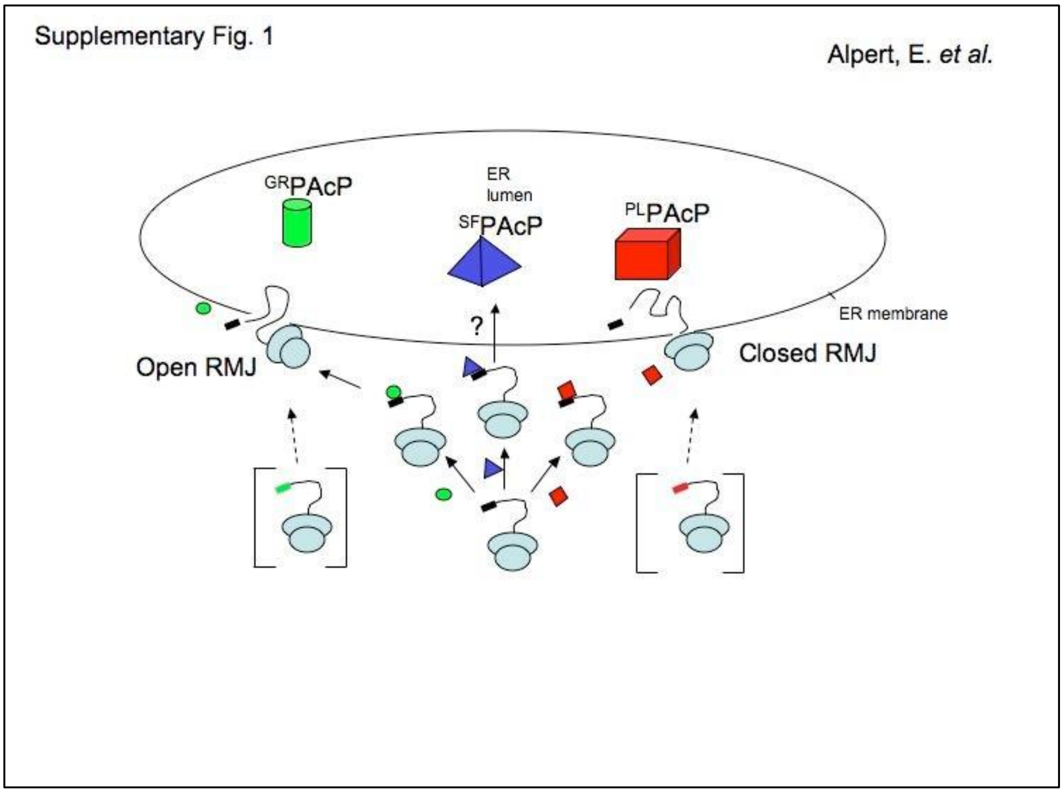

Supplementary Figure 1. PAcP biogenesis and the use of signal sequence swapping as a tool for protein subset enrichment. Information from previous studies[14,15,50] is utilized to formulate the working hypothesis that drives the present study. In brief, it is assumed that the nascent PAcP chain (bottom center) can naturally interact via its native signal sequence with three hypothetical *trans*-acting factors (indicated by green circle, blue triangle, and red square). One leads to formation of an open RMJ (left hand pathway), facilitating the folding pathway by which ^{GR}PAcP is achieved, by analogy to previous work[14,15]. Another forms a closed RMJ by which ^{PL}PAcP is formed (right hand pathway), again by analogy to previous studies[14,15]. The pathway and mechanism by which ^{SF}PAcP is formed (center pathway, leading to the blue triangle as the hypothesized *trans*-acting factor), could be distinct from those involving the

RMJ[14,50]. Regardless of their precise mechanism, each of these distinct pathways of biogenesis involves different folding funnels and final mature PAcP folded states (depicted as different colored shapes, green cylinder vs blue pyramid vs red cube in the ER lumen), and/or different PTMs. By using the IgG signal sequence domains (left hand brackets), regulation of that pathway by the trans-acting factor (green circle) is bypassed , avoiding regulatory mechanisms that might attenuate the robustness of flow of chains through the pathway (e.g. issues such as feedback downregulation, dependence on signalling pathway activation). Likewise, the Prl signal sequence domains (right hand brackets), allow bypass of the regulated pathway involving a different hypothesized *trans*-acting factor (red square) leading to the form of PAcP depicted as a red cube. Thus use of these heterologous signal sequences is proposed to skew distribution of the protein in favor of one or another subset. Because they bypass the physiological regulatory mechanisms that govern biogenesis pathway choice, the IgG and Prl signal sequences can be though of as acting in a "constitutive" rather than regulated manner. Like the signal sequence of PrP, that of PAcP may be termed a "regulated" signal sequence because it is capable of achieving either an open or closed RMJ, depending on the balance of active *trans*-acting factors present[14,15] . In the case of PrP one of the three pathways of biogenesis identified appears to be a default pathway[13]. Since untransfected LNCaP C-81 cells are androgen-independent and are not expressing PAcP, it is possible that the pathway(s) leading to ^{GR}PAcP and ^{SF}PAcP are default, constitutive pathways, while that leading to ^{PL}PAcP is tightly regulated. By loose analogy to a hormone "clamp" experiment, signal sequence swapping allows levels of a particular functional form of a protein (in this case PAcP), to be maintained without feedback downregulation that would occur physiologically. A signal sequence that bypasses the blue triangle *trans*-acting

factor to access that pathway has not yet been identified, and indeed the mechanism by which ^{SF}PAcP is achieved remains to be established.

Supplementary Fig. 2

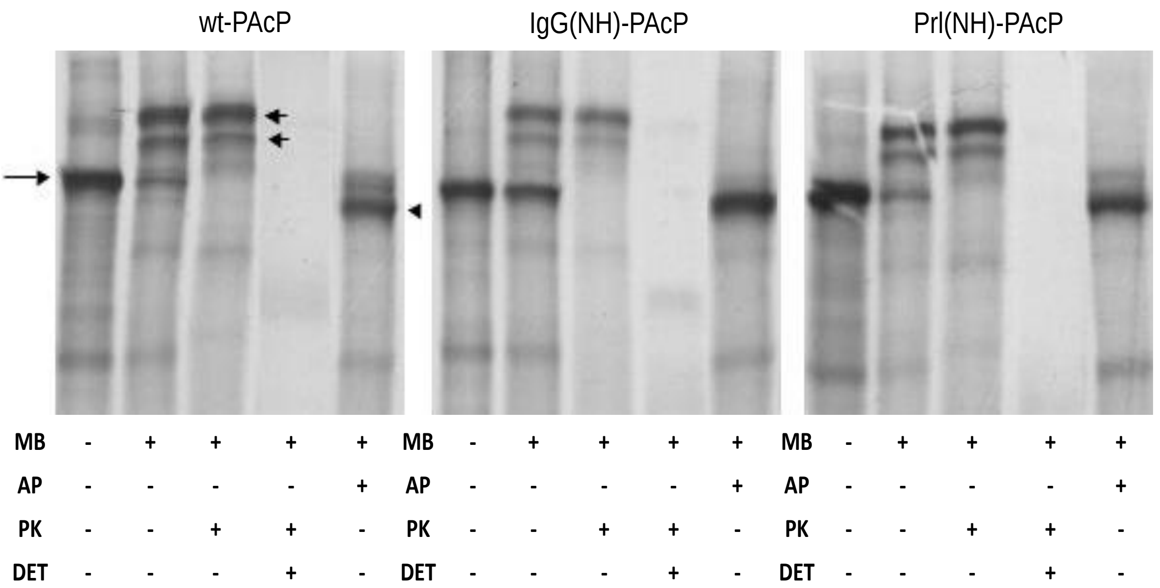

Supplementary Figure 2. Transcription-linked translation of PAcP. Cell- free protein synthesis was carried out using the rabbit reticulocyte lysate translation system as previously[51] and programmed with PAcP and the signal sequence chimeric constructs engineered behind the SP6 promoter. Products were synthesized in the presence or absence of microsomal membranes from dog pancreas to study translocation to the ER lumen and glycosylation[52]. In some cases acceptor peptide (AP) was included to block glycosylation and confirm signal sequence cleavage by migration, as previously reported[53].

Selected samples were treated with proteinase K (PK) at a final concentration of

0.25mg/ml in the presence or absence of non-denaturing detergent as previously reported[51], and analyzed by SDS PAGE and autoradiography (AR). Note protection from PK in the presence of microsomal membranes (MB) but in the absence of detergent (DET), of the glycosylated bands but not of the precursor band, as evidence of translocation to the ER lumen, as previously reported[51].

Significance of Supplementary Fig. 2: This data demonstrates that the chimeric signal sequences composed of the N and H domains from the IgG and Prl signal sequences engineered upstream of the PAcP signal sequence C domain coding region function in a manner indistinguishable from the wtPAcP signal sequence for efficiency and outcome of targeting, signal sequence cleavage, and translocation across the ER membrane. What is not being monitored is the folding and function of the protein behind these different signal sequence. The manuscript demonstrates remarkable changes in folding and function of PAcP in response to swapping the signal squene domains.

Supplementary Fig. 3

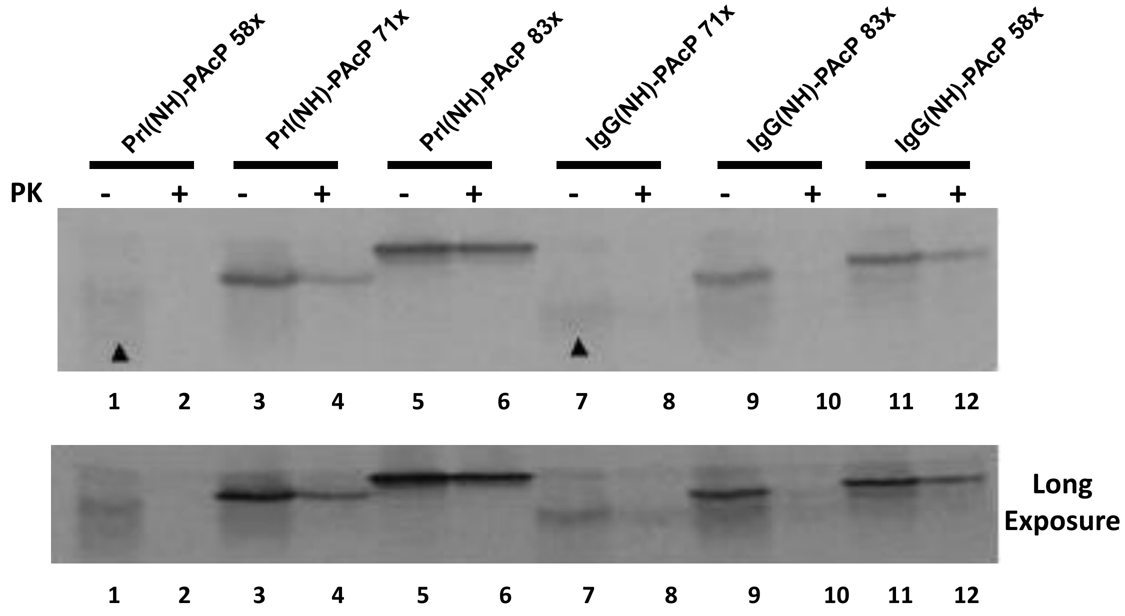

Supplementary Figure 3. Characterization of the PAcP RMJ formed behind chimeric signal sequences. As shown by the arrow heads in lanes 1 and 7,

short polypeptide chains which are expectedly untargeted are largely excluded from analysis. Of the small number that are targeted at the shortest length, neither signal sequence has established a PK-resistant RMJ. At longer chain lengths, both Prl(NH)-PAcP and IgG(NH)-PAcP were targeted and became progressively more resistant to PK treatment. However, at similar chain lengths, IgG(NH)-PAcP polypeptides were consistently more accessible to proteolysis. The most dramatic difference is shown in the case of IgG(NH)-PAcP$_{83X}$ (lane 9/10) which is completely PK digested (shown appreciably by a longer exposure in the lower panel) although a prominent fraction of Prl(NH)-PAcP$_{71X}$ is protected from proteolysis (lane 3/4). These results confirm that IgG signal sequence domains on PAcP form a relatively more “open” RMJ than do the corresponding domains from the Prl signal sequence, as observed previously [14,15] where those differences were correlated to different pathways of biogenesis at the ER.

Significance of Supplementary Fig 3: This figure demonstrates that the IgG signal sequence creates an “open” RMJ while that of Prl forms a “closed” RMJ when engineered onto PAcP. Hence providing a rationale for choice of those two signal sequences rather than any random others for purpose of signal sequence domain swapping.

Supplementary Fig. 4

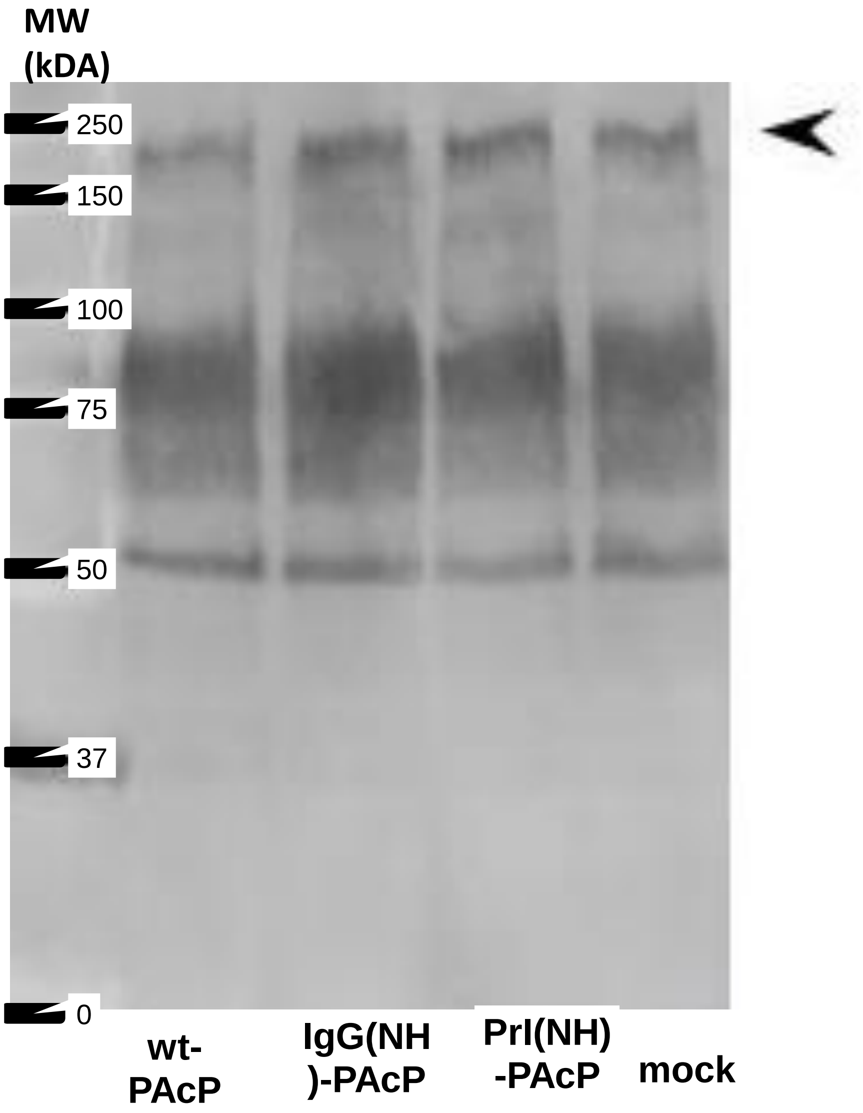

Supplementary Figure 4. Addition of Prl(NH)-PAcP medium restores phosphorylation of c-Erb-B2 (p185). As for Fig. 1E, except with medium replaced by that from Prl(NH)-PAcP transfected cells as in Fig. 1G instead of androgen supplementation.

Significance of Supplementary Fig. 4: Demonstrates that Prl(NH)-PAcP transfected cells secrete a growth factor that activates the ErbB-related signalling pathway as shown in manuscript Fig. 1D, but in the absence of androgen.

Supplementary Fig. 5

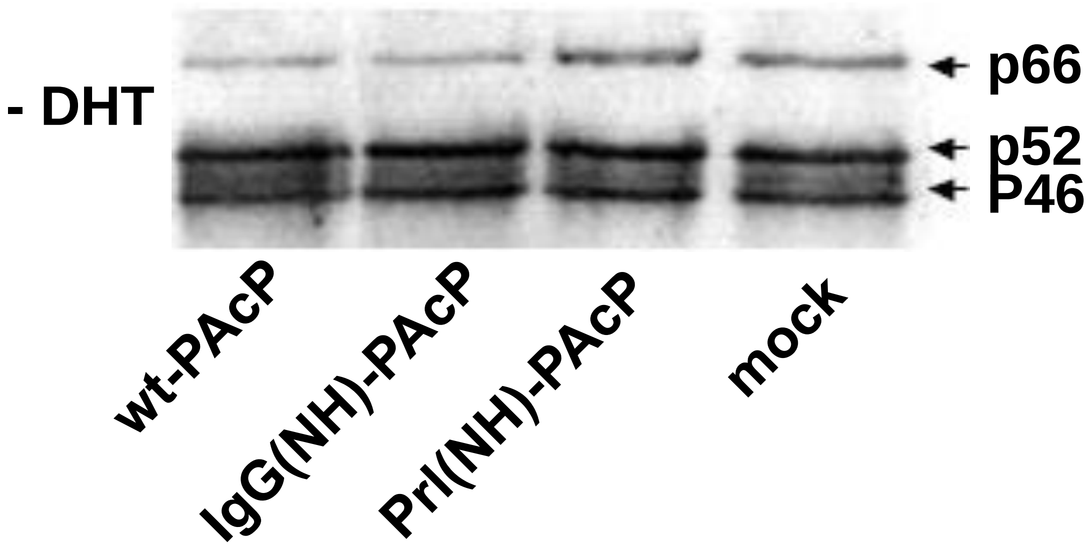

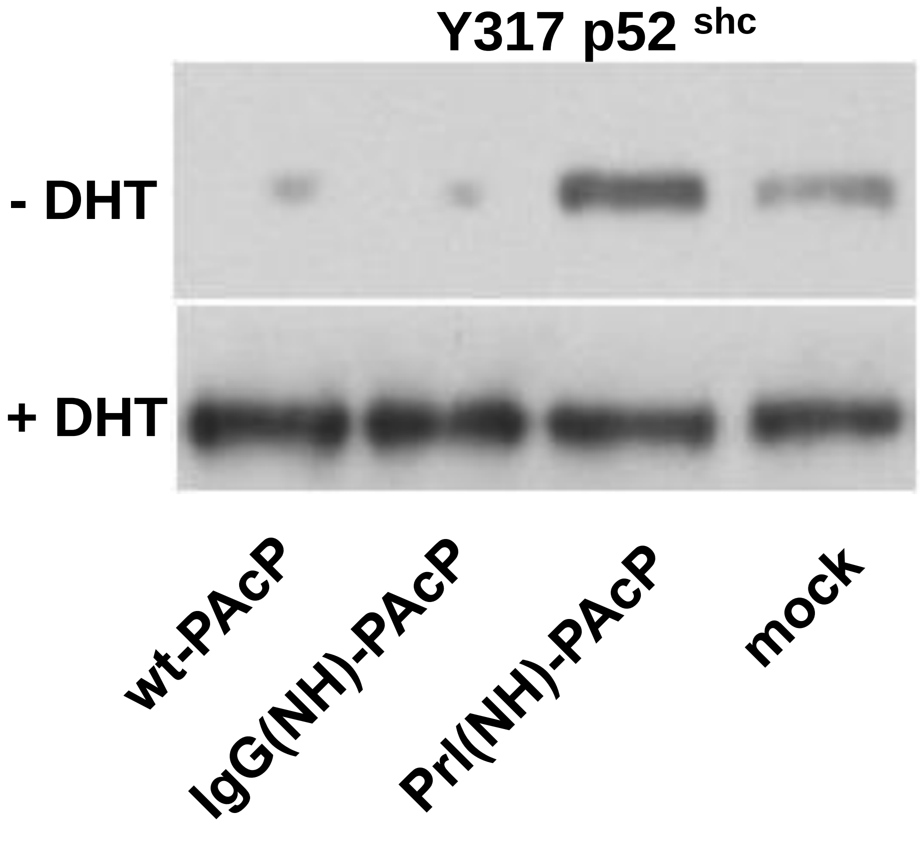

Supplementary Figure 5. Shc phosphorylation in transfected cells. Cells were transfected in the presence or absence of 10 nM DHT as described in Methods. 72 hours after transfection, cells were harvested, lysed and equal amount of total protein (50 µg) was electrophoresed on 10% SDS-PAGE for WB analysis with the anti-Shc antibody (upper panel). 1mg of total protein of each individual treatment was immunoprecipitated with 5 µg anti-Shc antibody (Upstate) and analyzed by WB with Y317p52 Shc antibody (lower panel). Note that the intensity of the p66 band is diminished in cells that have become androgen-dependent, i.e. wtPAcP and IgG(NH)- PAcP transfected cells (upper panel). When these same samples were immunoprecipitated with anti-Shc antibody and analyzed by WB with Y317 p52- specific antibody , androgen-dependent cells show lack of p52 Y317 phosphorylation in the absence of added androgen, and gain of p52 Y317 phosphorylation upon addition of DHT.

Significance of Supplementary Fig. 5: Phosphorylation of p52 on Y317 suggests that the phosphorylation of p185 shown previously activates a functional phosphorylation cascade resulting in androgen-dependent growth.

Alpert, E. *et a*

Supplementary Fig. 6A

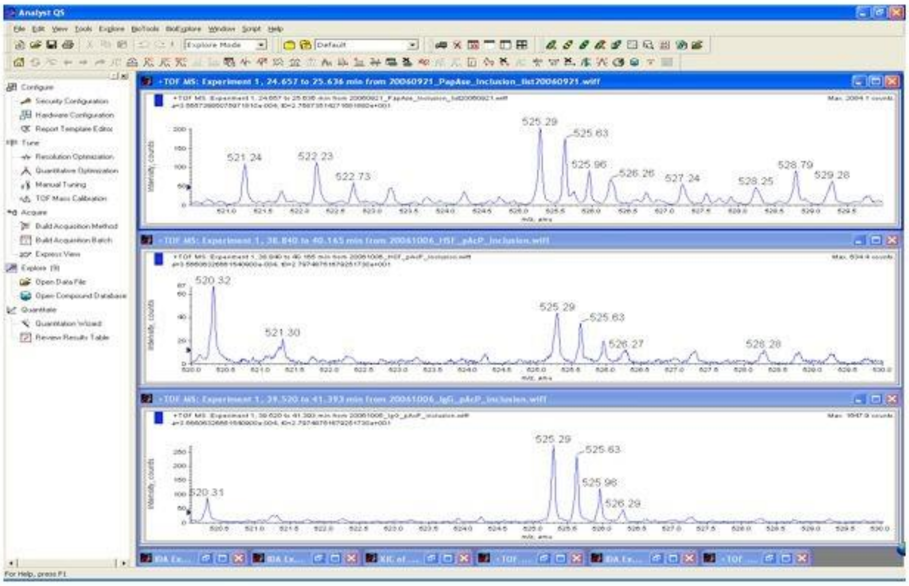

1

Alpert, E. *et al.*

Supplementary Fig. 6B

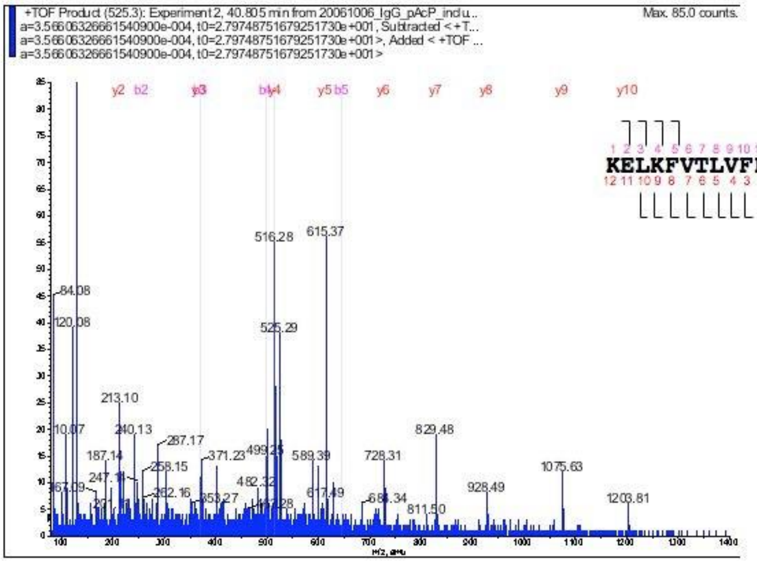

Supplementary Fig. 6. Mass spectrometry of Prl(NH)PAcP and IgG(NH)PAcP. (**A**) **LC MS spectra of the triply charged molecular ion of the AspN peptide spanning position 1- observed in three different samples of PAcP**. Top panel – Prl(NH)-PAcP from media, middle panel – PAcP from HSP, lower panel – IgG(NH)- PAcP from cellular fraction. Analyses of HSP and IgG(NH)-PAcP were performed on the same day; Prl(NH)-PAcP was analyzed on separate date utilizing different LC column. (**B**). **Electrospray ionization MS/MS spectrum of the peptide KELKFVTLVFRHG (D)**. Fragment ions of *b*- and *y*-series are annotated.

Significance of Supplementary Fig. 6: This data demonstrates the primary sequence identity of ^{GR}PAcP, ^{PL}PAcP, and ^{SF}PAcP, setting the stage for further characterization of the structural differences between these functionally different subsets of PAcP.

Supplementary Fig. 7A

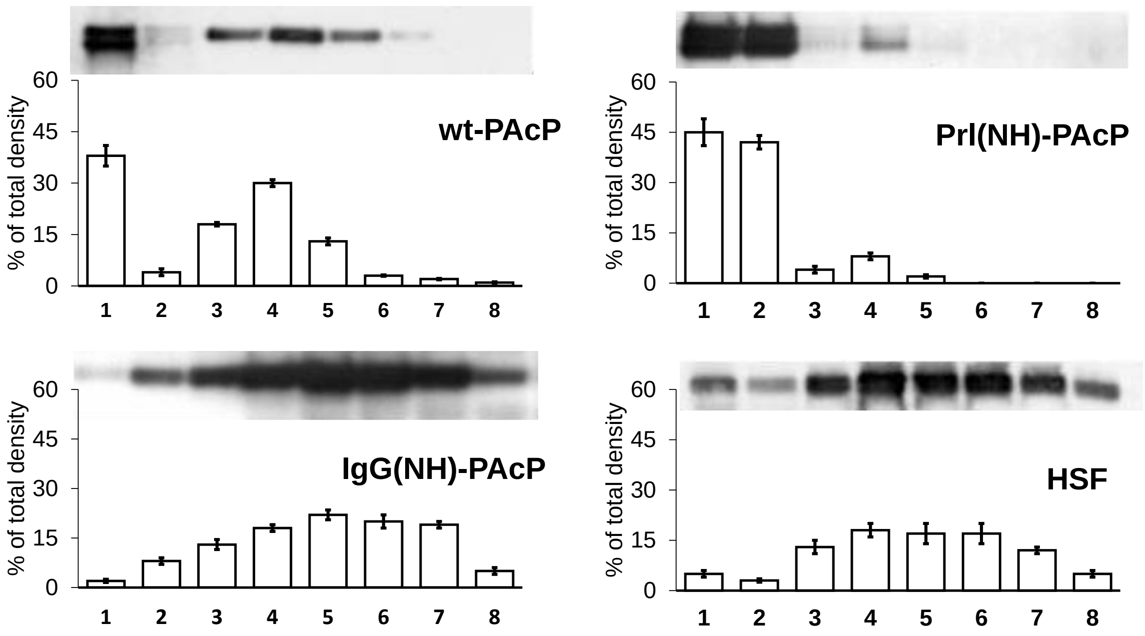

Supplementary Fig. 7B

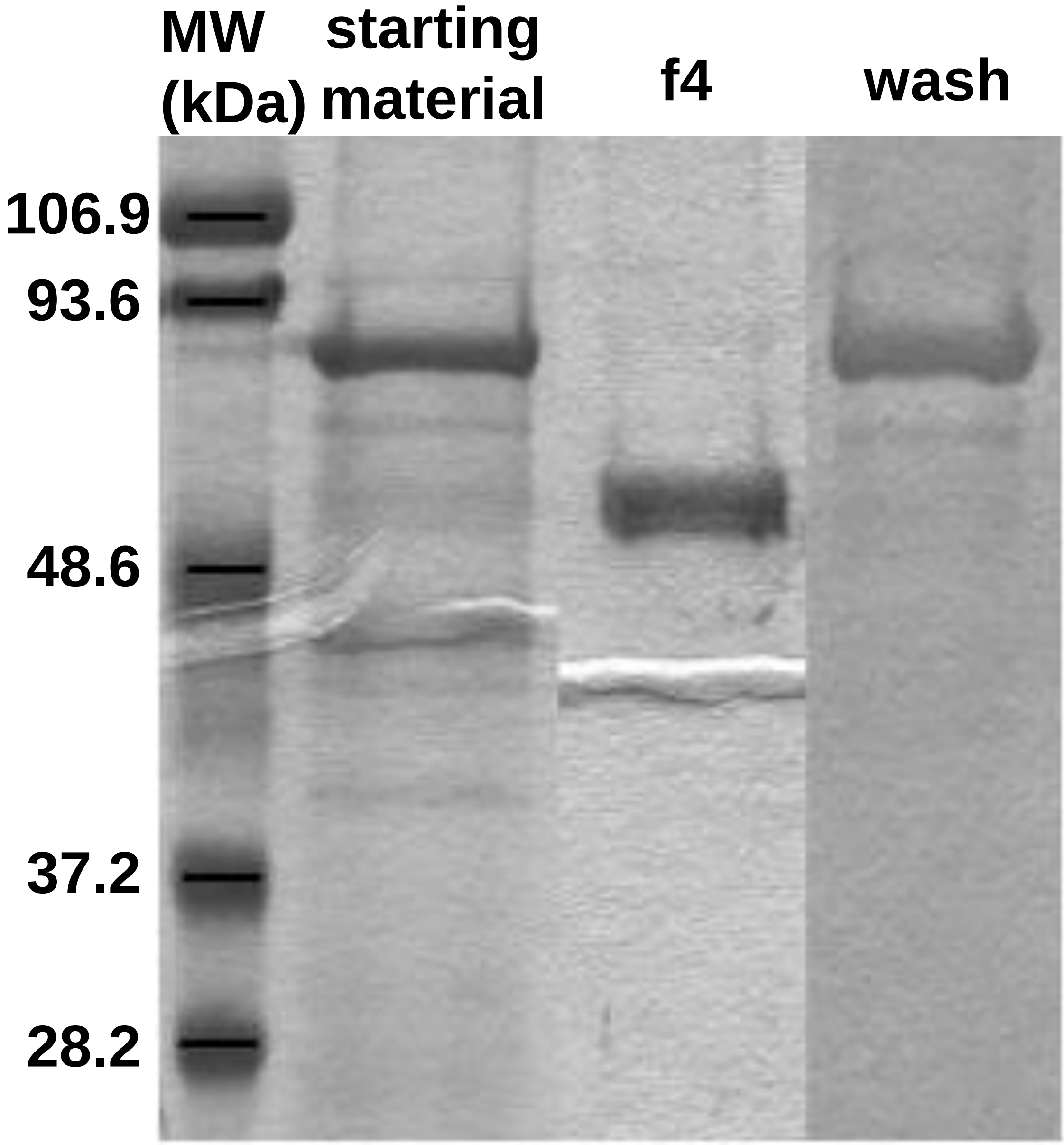

Supplementary Figure 7. Affinity chromatography of PAcP. Coupling of L(+)-tartrate was carried out as described[36]. The column containing linked L(+)-tartrate was equilibrated with sodium acetate buffer (50 mmol/liter, pH 5.0). Cell lysates of LNCaP clone C 81 transfected cells with indicated signal sequences and HSP (the amount of PAcP was first normalized by WB) were applied onto the column.

The enzyme was then eluted by linear gradient of the same buffer containing from 0 to 10 mM of L(+)-tartrate. A flow rate was 26 ml/h. Fractions were dialyzed against deionizated water overnight, then lyophilized. (**A**) **WB analysis**. Samples were analyzed by WB using anti-PAcP antiserum. Left: 1 – 8 represents fractions number. Fractions 9 and 10 were empty (data not shown). Graph shown is % of distribution

from total density. Data shown are mean±S.D. (n=3), $p<0.05$. (**B**) **Coomassie blue stain.** For the stain 1/100 of the starting material or 1/10 of dialysed fraction number 4 or 1/10 of dialysed wash (fraction 2) of HSP was used.

Significance of Supplementary Fig. 7: L(+)-tartrate affinity chromatography provided another line of evidence for PAcP heterogeneity and the utility of signal sequence swapping to magnify subsets of the forms of PAcP.

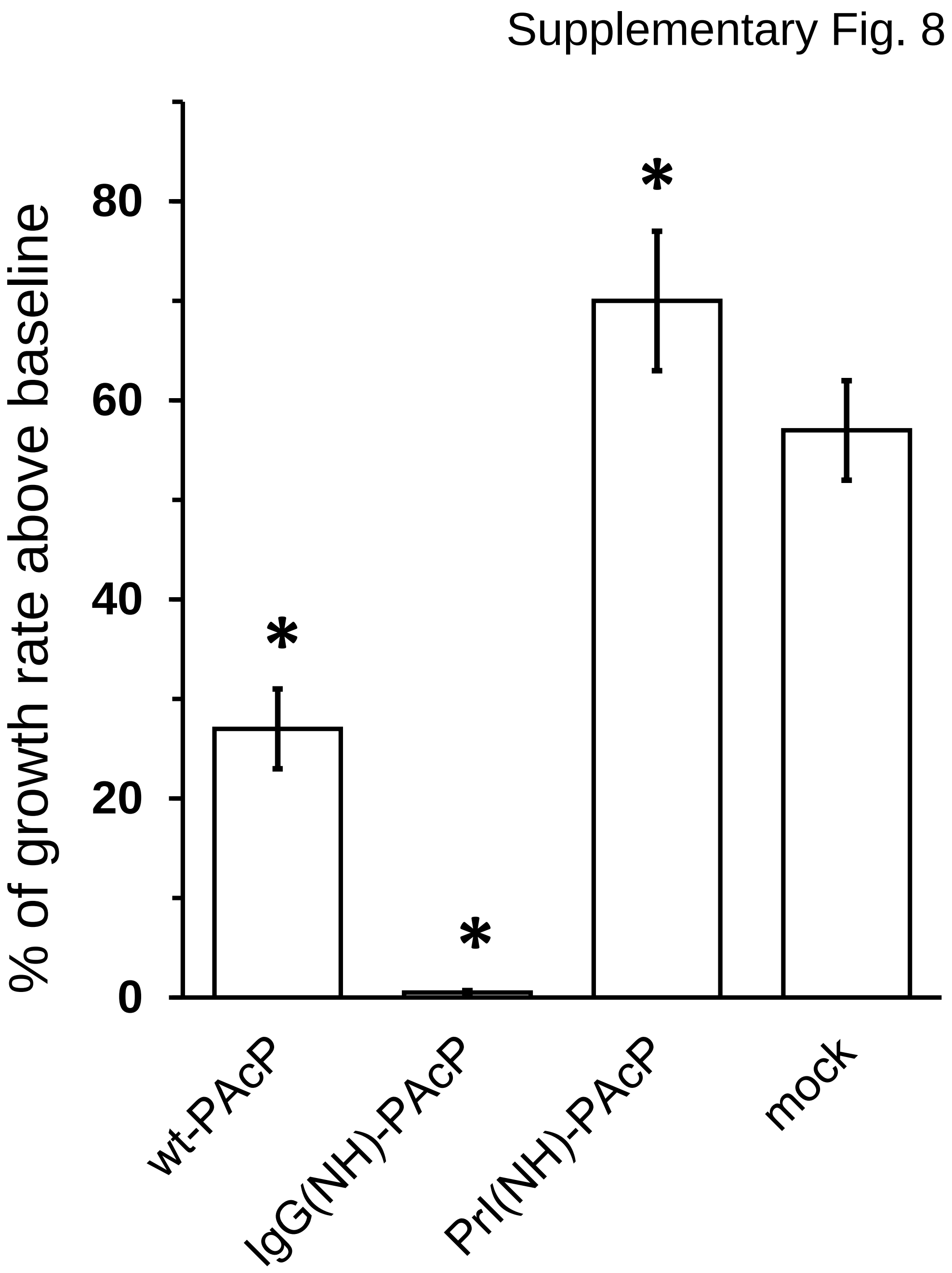

Supplementary Figure 8. Addition of wtPAcP transfected cell medium does *not* overcome androgen-dependence for growth of IgG(NH)PAcP transfected cells. Two sets of LNCaP clone C 81 cells were transfected with wt PAcP or empty vector. 72 hours after transfection media from both sets was harvested and used as a replacement medium for another two sets of cells upon completion of transfection as indicated (5 hours). 72 hours later, cells were harvested and counted. Baseline for calculation of percent growth was taken to be growth in the presence of C81 (mock transfection) medium. A control set of cells was transfected as indicated at the same time without

replacement media. The number of cells in that control was not statistically significantly different from plate that received mock replacement medium. Data shown are % of growth rate above baseline. Mean±S.D. (n=4) *p<0.01, significantly different from control

Significance of Supplementary Fig. 8: This demonstrates that PAcP in the medium from wtPAcP transfected cells is different in function from PAcP in medium from Prl(NH)-PAcP transfected cells, corroborating the prediction from L(+)-tartrate inhibition studies (Fig. 3C of manuscript) and 2DGE (Fig. 5) that failed to reveal ^{PL}PAcP in either cells or medium from wtPAcP-transfected LNCaP C-81 cells. Instead, wtPAcP cells contain ^{GR}PAcP and medium contains ^{SF}PAcP. This suggests these are default pathways because they are present in the LN CaP C-81 cell line that is no longer expresses PAcP.

Supplementary Table 1. Structural Properties of PAcP Conformers

Alpert, E. *et al.*

	^{GR}PAcP	wtPAcP cell	wtPAcP medium	^{PL}PAcP	^{SF}PAcP	Early Prostate Cancer
Immunoreactivity after biotinylation						
nat	-	+/-	+/-	+	nd	nd
den	+	+	+	+	nd	nd
Tartrate Sens/Resist	S	S/R	nd	R	S	nd
Tartrate column Peak	4-6	1,4		1,2	4-6	nd
Trypsin						
frag pattern	1	3	nd	2	nd	nd
resistance	+	+/-	nd	-	nd	nd
Distinctive 2D gel spots	#1-3	#1-3	#2, #4	#5	#2, #4	#5

References used only in the "Supplementary Materials"

50. Ott CM, Lingappa VR. Signal sequences influence membrane integration of the prion protein. Biochemistry. 2004;43(38):11973-82.
51. Shields D, Blobel G. Efficient cleavage and segregation of nascent presecretory proteins in a reticulocyte lysate supplemented with microsomal membranes. J. Biol. Chem. 1978;253(11):3753-6.
52. Lingappa VR, Lingappa JR., Prasad R, Ebner KE, Blobel, G. Coupled cell-free synthesis, segregation, and core glycosylation of a secretory protein. Proc Natl Acad Sci U S A. 1978;75(5):2338-42.
53. Skach WR, Calayag MC, Lingappa VR. Evidence for an alternate model of human P-glycoprotein structure and biogenesis. J. Biol. Chem. 1993;268(10):6903-08.
